# Metabolism of cancer cells and immune cells in the initiation, progression, and metastasis of cancer

**DOI:** 10.7150/thno.103376

**Published:** 2025-01-01

**Authors:** Mingxia Jiang, Huapan Fang, Huayu Tian

**Affiliations:** 1 State Key Laboratory of Physical Chemistry of Solid Surfaces, College of Chemistry and Chemical Engineering, Innovation Laboratory for Sciences and Technologies of Energy Materials of Fujian Province (IKKEM), Xiamen University, Xiamen 361005, China.; 2 Shenzhen Research Institute of Xiamen University, Shenzhen 518000, China.

**Keywords:** Metabolism of cancer and immune cells, Metabolic reprogramming, Cancer niches, Anti-tumor immune responses, Cancer immunotherapy

## Abstract

The metabolism of cancer and immune cells plays a crucial role in the initiation, progression, and metastasis of cancer. Cancer cells often undergo metabolic reprogramming to sustain their rapid growth and proliferation, along with meeting their energy demands and biosynthetic needs. Nevertheless, immune cells execute their immune response functions through the specific metabolic pathways, either to recognize, attack, and eliminate cancer cells or to promote the growth or metastasis of cancer cells. The alteration of cancer niches will impact the metabolism of both cancer and immune cells, modulating the survival and proliferation of cancer cells, and the activation and efficacy of immune cells. This review systematically describes the key characteristics of cancer cell metabolism and elucidates how such metabolic traits influence the metabolic behavior of immune cells. Moreover, this article also highlights the crucial role of immune cell metabolism in anti-tumor immune responses, particularly in priming T cell activation and function. By comprehensively exploring the metabolic crosstalk between cancer and immune cells in cancer niche, the aim is to discover novel strategies of cancer immunotherapy and provide effective guidance for clinical research in cancer treatment. In addition, the review also discusses current challenges such as the inadequacy of relevant diagnostic technologies and the issue of multidrug resistance, and proposes potential solutions including bolstering foundational cancer research, fostering technological innovation, and implementing precision medicine approaches. In-depth research into the metabolic effects of cancer niches can improve cancer treatment outcomes, prolong patients' survival period and enhance their quality of life.

## 1. Introduction

Cancer, as a primary threat to human life and health, imposes a heavy burden on patients' families and society 1-4. According to the updated estimates from the International Agency for Research on Cancer (IARC) in 2022, there were nearly 20 million new cancer cases and 9.7 million cancer-related deaths worldwide 5. The incidence and mortality rates of cancer often vary depending on the type of cancer, region, age, gender, lifestyle, or other factors 6. Cancer, referred to as a malignant tumor, is a disease caused by the uncontrolled proliferation of aberrant cells in the body 7, and these deviant cells have the capacity to disseminate throughout the body via the lymphatic system, blood circulation, and direct infiltration of surrounding tissues, thereby compromising the body's immune system and culminating in multiple organ dysfunction 8.

Cancer is a multifaceted disease characterized by aberrant growth and invasive spread of cancer cells, sustained by a cancer niche influenced by the communication of a variety of cells, especially cancer cells and immune cells 9, 10. In this niche, the metabolic reprogramming of cancer cells, and subsequently, the adaptive metabolic response of immune cells has profound effects on the occurrence, growth, and metastasis of cancers 11, 12. Moreover, the cancer niches undergoes substantial alterations during the occurrence and development of cancer 13, which significantly influence on the growth, proliferation, and immune evasion of cancer cells 14. Furthermore, elucidating the metabolism processes of cancer cells and immune cells in the cancer niche plays a crucial role in oncological research and the design of treatment formulations 15.

**Figure 1:**
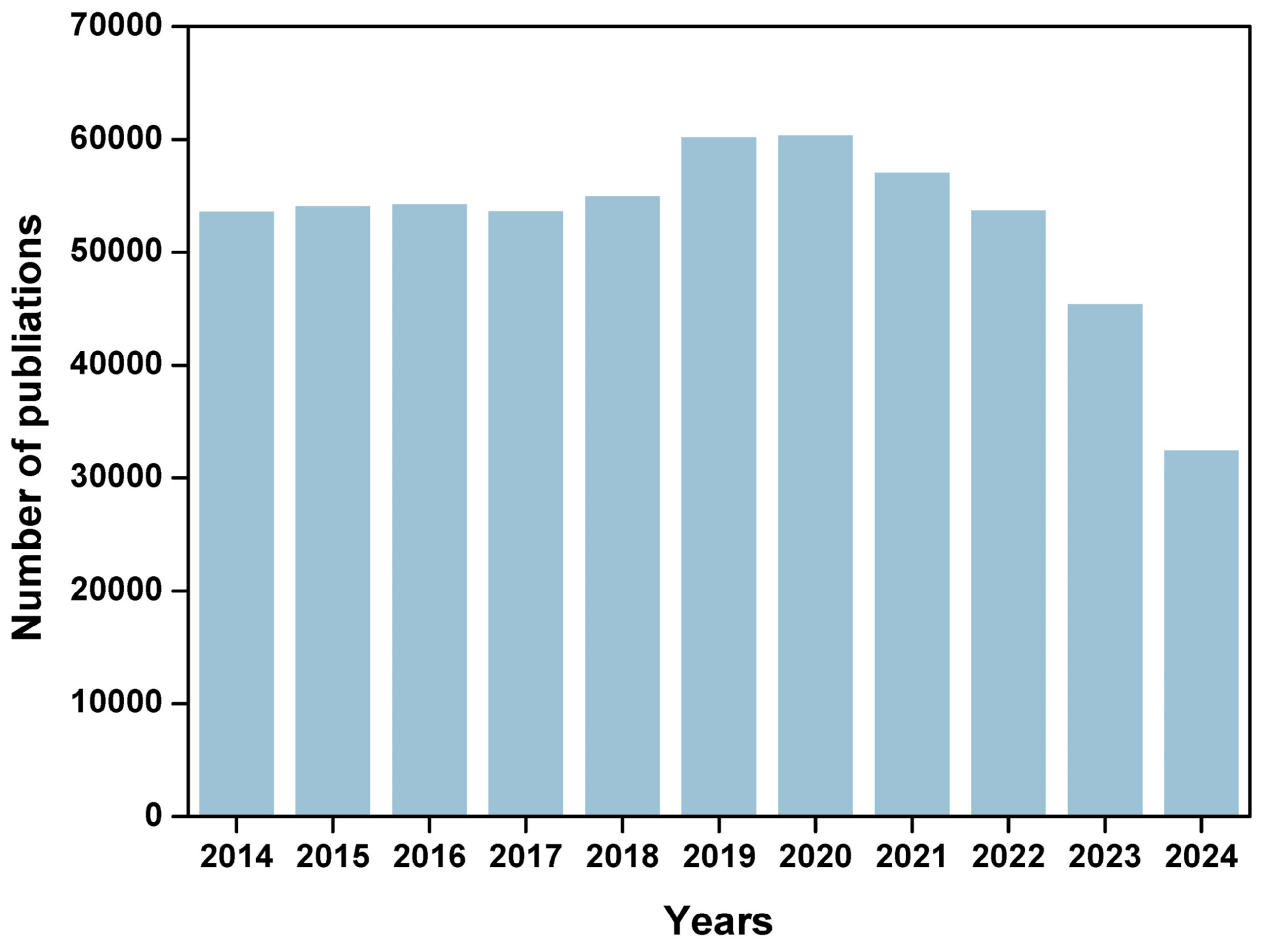
Number of publications by searching “cell metabolism and cancer” from 2014 to 2024 in Web of Science (The latest data of 2024 was acquired on Sep. 2024).

Cancer cells utilize metabolic reprogramming to sustain the energy and biosynthetic materials needed for their rapid proliferation and dissemination 16. This reprogramming also alters the metabolic state of the cancer niche, further influencing the function of immune cells. For example, cancer cells can leverage amino acid metabolism and lipid metabolism to enhance their proliferative activity and environmental adaptability 17. However, immune-active cells such as CTLs cells and NK cells often face metabolic and nutrient competition with cancer cells in the cancer niches 18. It is essential to regulate the immunometabolic pathways of cancer and immune cells to enhance anti-tumor immune effects. Understanding the metabolic mechanisms of cancer cells and immune cells will provide robust guidance for cancer immunotherapy. Targeting cancer cell metabolic pathways or modulating the metabolic state of immune cells can help activate the immune system to recognize and eliminate cancer cells, thereby exerting anti-tumor effects 19, 20. Therefore, comprehensively exploring the metabolic mechanisms between cancer cells and immune cells are crucial for understanding the role of immune cells in cancer progression and developing targeted interventions. As shown in **Figure 1**, according to Web of Science statistics, the number of articles on “cell metabolism and cancer” has exceeded 40000 in the past decade. These research areas have shown important potential in improving the efficacy of cancer treatment and optimizing immunotherapy strategies.

**Figure 2:**
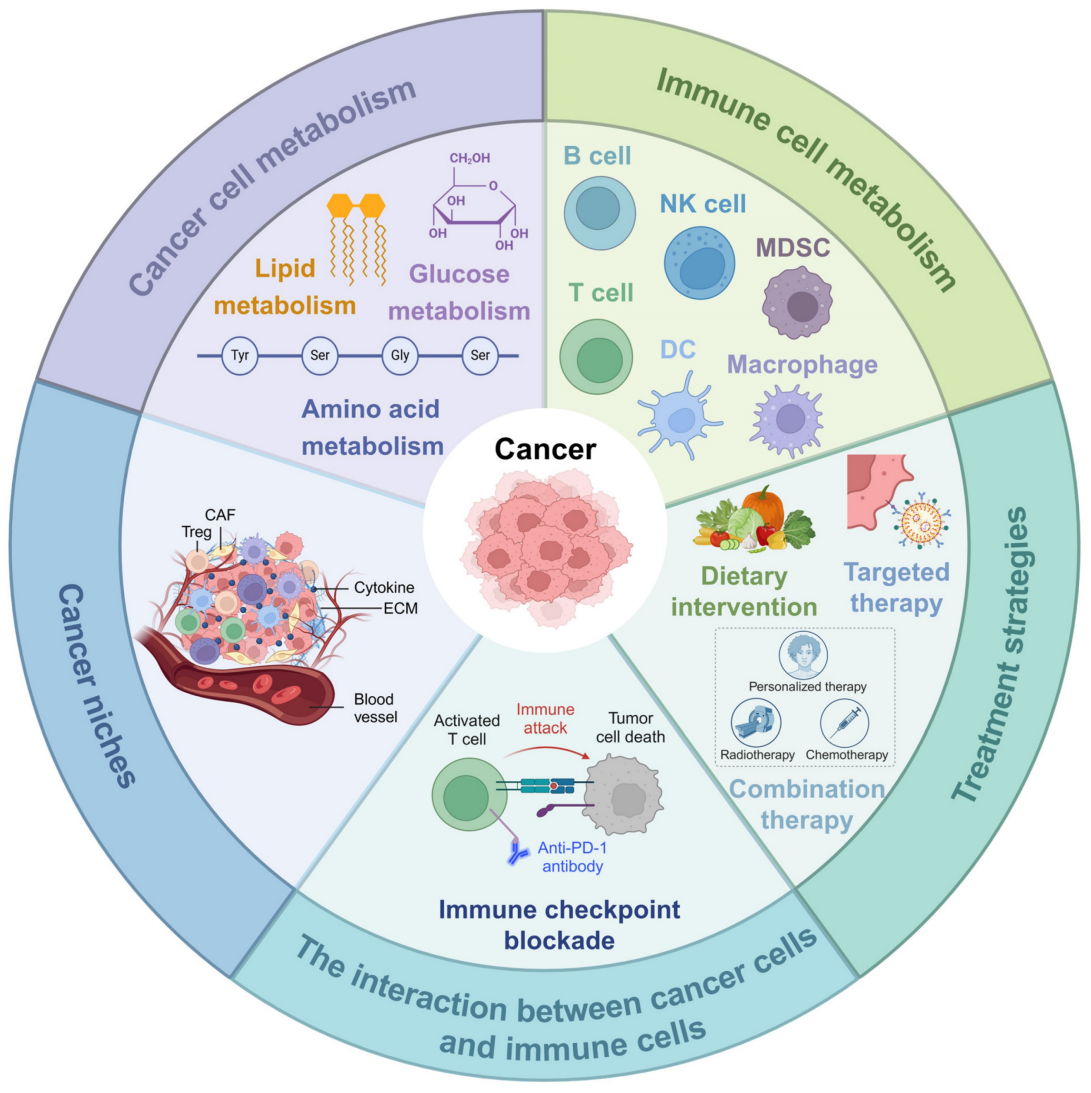
Metabolism of cancer and immune cells. NK, nature killer; DC, dendritic cell; MDSC, myeloid-derived suppressor cells; CAF, cancer-associated fibroblast; ECM, extracellular matrix. This figure was created with Biorender.com.

In this review, we delve into the role of metabolism in cancer development, growth, and metastasis, reviewing the latest research advancements in the metabolism of cancer cells and immune cells for cancer therapy. Moreover, we highlight the opportunities and challenges faced by current metabolic intervention strategies and propose future research directions and therapeutic strategies based on metabolic pathways (**Figure 2**). This review aims to provide new insights and methods for clinical cancer treatment through regulating metabolic pathways.

## 2. Cancer cell metabolism

Cancer cell metabolism holds significant importance within the field of oncology, tracing its origins back to the pioneering research of Otto Warburg. Otto Warburg first discovered the unique metabolic phenomenon of cancer cells in the early 20^th^ century 21. Otto Warburg identified a distinctive metabolic characteristic of cancer cell wherein they exhibit a preference for glycolysis over oxidative phosphorylation for energy generation, even in oxygen-rich environments. The metabolic phenomenon, commonly referred to as the “Warburg effect”, continues to be a focal point of investigation in cancer research.

**Figure 3:**
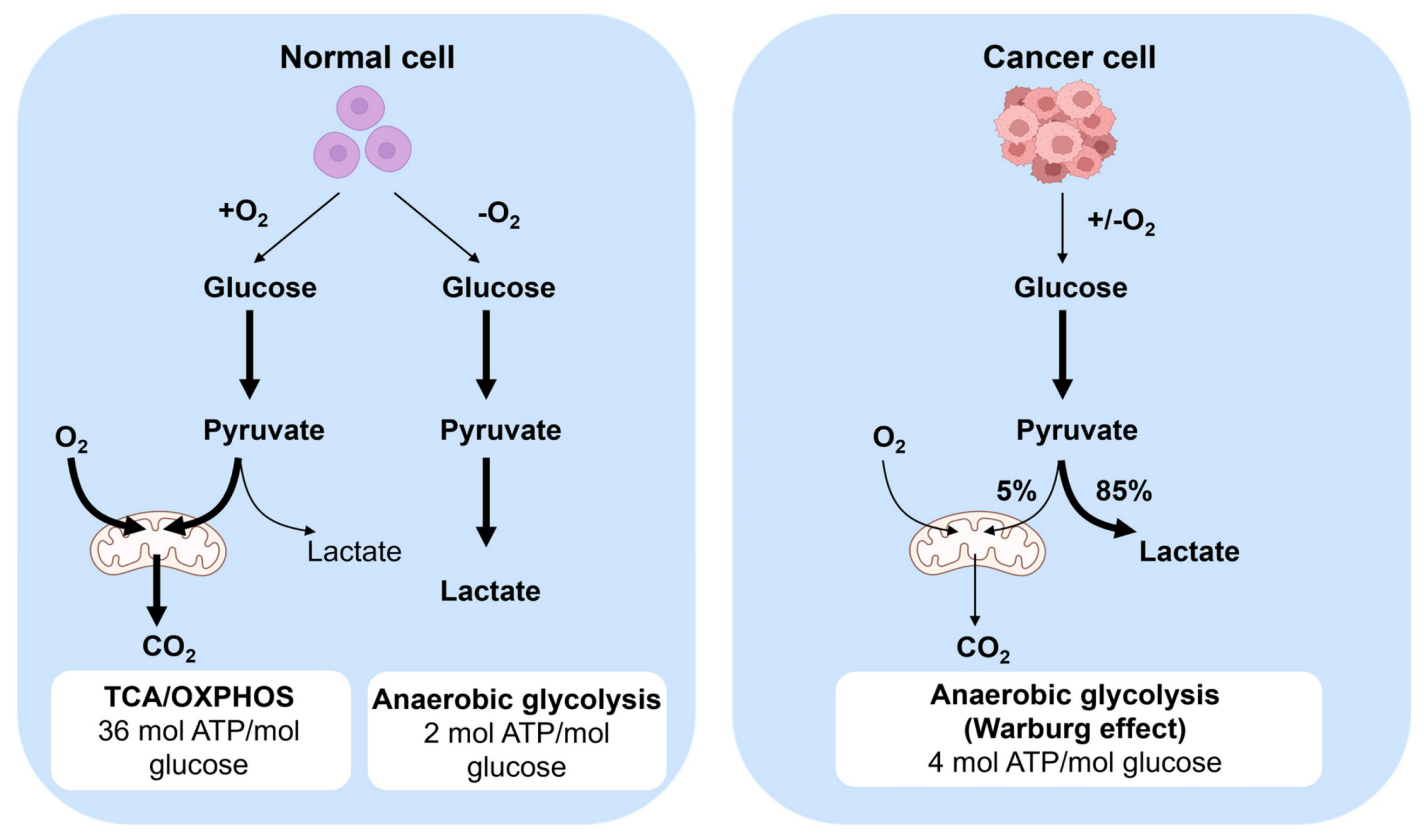
Metabolism in normal and cancer cells. In normal cells, one of two pathways is utilized. When oxygen is present, glucose is converted to pyruvate and subsequently followed by mitochondrial oxidation of pyruvate to carbon dioxide by entering into the tricarboxylic acid (TCA) cycle and the oxidative phosphorylation process which produces 36 ATPs per glucose. When no oxygen is present, glucose is metabolized to lactate, which yields 2 ATPs per glucose. Cancer cell convert the majority of glucose to lactate to yield 4 ATPs per glucose, even in the presence of oxygen. This is called the Warburg Effect. Glucose metabolites are converged from energy generation to anabolic process to increase cell proliferation.

The metabolic behavior of cancer cells diverges from that of normal cells **(Figure 3)**, with cancer cell exhibiting distinct characteristics in energy production 22. While normal cells predominantly rely on aerobic metabolism in the presence of oxygen, cancer cells tend to favor anaerobic glycolysis even in oxygen-rich conditions to generate energy. The specific process involves converting glucose to pyruvate, followed by oxidative phosphorylation in mitochondria, and ultimately converting it to carbon dioxide and producing more adenosine triphosphate (ATP) 21. This metabolic pathway leads to cancer cells displaying a heightened glucose consumption rate compared to normal cells. Moreover, cancer cells excrete lactate produced during this process outside the cells to prevent substrate accumulation. Despite the elevated energy demand of cancer cells, their glycolytic metabolism is relatively inefficient in ATP production than the oxidative phosphorylation pathway utilized by normal cells. Furthermore, cancer cells undergo metabolic changes to support their rapid growth and proliferation, such as enhancing synthetic anabolism by modifying their metabolic pathways to increase the synthesis of amino acids, nucleotides, and lipids 23. Additionally, cancer cells can also release toxins and alter the metabolic environment within the body, thereby facilitating tumor progression and metastasis.

### 2.1. Metabolic characteristics of cancer cells

The metabolic processes of cancer cells are intricately linked to metabolic reprogramming, which involves the adjustment of metabolic pathways and products to accommodate the rapid growth, unlimited proliferation, and invasion of cancer cells during tumor progression 24, 25. Metabolic reprogramming of cancer cells is considered a major hallmark of cancer. Cancer cells undergo a series of metabolic reprogramming during their growth and proliferation, enabling them to adapt to their rapid growth and proliferation requirements.

#### 2.1.1. Glucose metabolism

**Figure 4:**
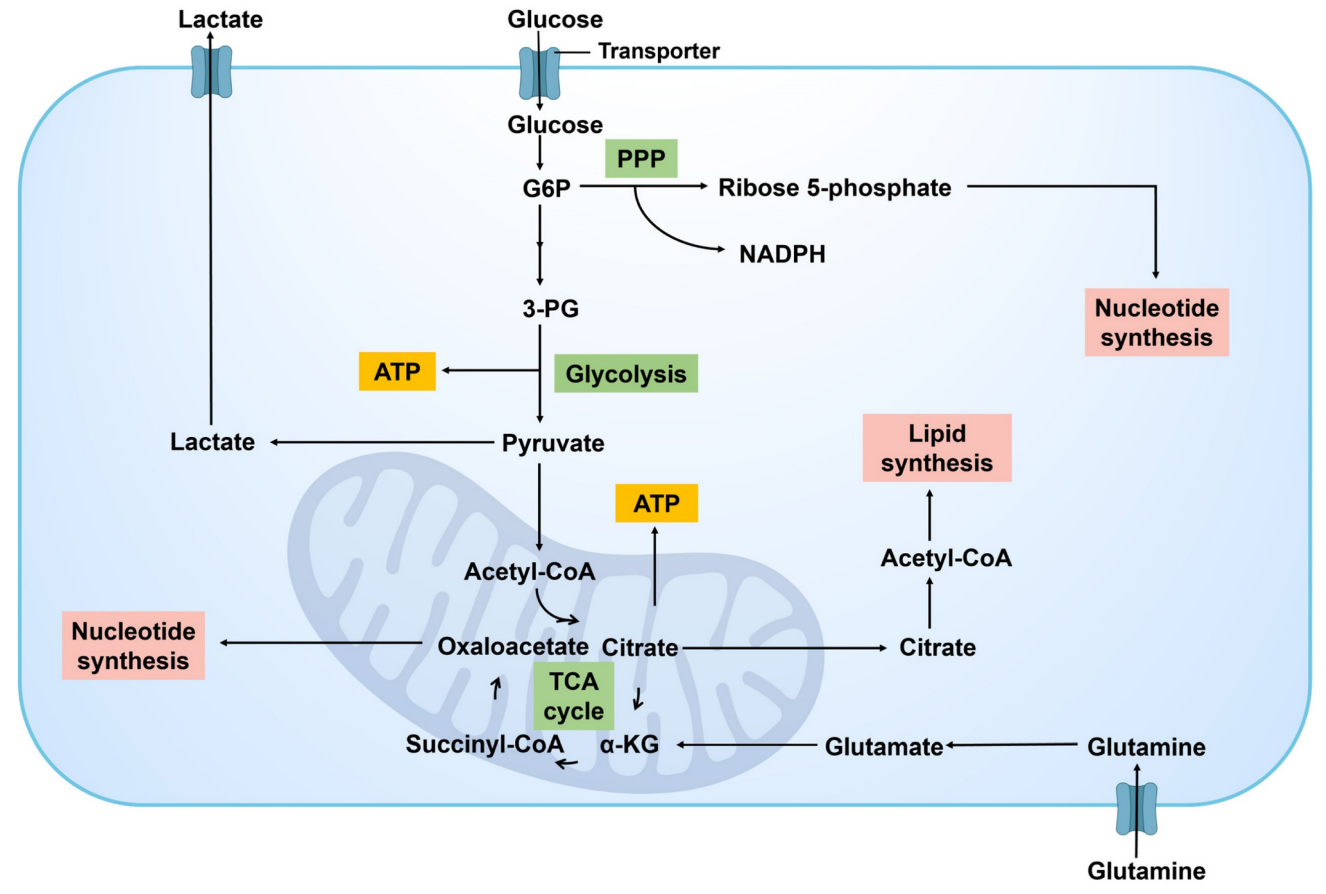
Glucose metabolism in cancer cells. G6P, glucose 6-phosphate; NADPH, nicotinamide adenine dinucleotide phosphate hydrogen; PPP, pentose phosphate pathway; 3-PG, 3-phosphoglycerate; ATP, triphosphate; α-KG, α-ketoglutarate.

Glucose metabolism is a pivotal aspect of the metabolic reprogramming observed in cancer cells, which is reflected in its adaptation to the need for energy supply and biosynthetic substances **(Figure 4)**. Glucose undergoes conversion into pyruvate via glycolysis, followed by the transformation of pyruvate into acetyl coenzyme A (Acetyl-CoA), which is subsequently oxidized in the tricarboxylic acid (TCA) cycle to generate ATP. An alternative pathway involves the conversion of pyruvate to lactic acid by lactate dehydrogenase (LDH), serving as the final product of glycolysis.

A significant feature of metabolic reprogramming in cancer cells is the Warburg effect 26. Cancer cells exhibit a preference for utilizing glycolysis over aerobic respiration for energy production, even in the presence of sufficient oxygen, resulting in the accumulation of significant quantities of lactic acid. This efficient glycolysis process not only provides the necessary ATP for the rapid proliferation of cancer cells but also facilitates cellular growth and division by furnishing precursor molecules for biosynthetic pathways 27, such as intermediates for the biosynthesis of nucleotides, amino acids, and lipids. It can be posited that cancer cells rely heavily on glycolysis. Alongside the heightened glycolytic activity, alterations in glucose metabolism in cancer cell encompass modifications in the pentose phosphate pathway (PPP), the TCA cycle, and related metabolic pathways. The PPP supplies cancer cells with both reducing power (NADPH, nicotinamide adenine dinucleotide phosphate hydrogen) and ribose, crucial for nucleotide synthesis 28, thereby supporting the rapid synthesis of DNA and RNA. At the same time, some key metabolic intermediates in glycolysis can also enter the TCA cycle to bolster energy production and provide molecules for biosynthesis 29.

In addition, cancer cells respond to their elevated metabolic demands and harsh microenvironment by engaging in a sequence of metabolic modifications, including “anaplerotic reactions” in various metabolic pathways. These modifications involve upregulating glucose uptake though the upregulation of glucose transporters (GLUTs) 30 and regulating specific metabolic pathways by altering the activity or expression of metabolic enzymes 31. These changes not only bolster the survival and proliferation capabilities of cancer cells but also enable them to acclimate to fluctuations in nutrient and oxygen levels within the microenvironment.

In addition, some key glycometabolism targets and signaling pathways play a crucial role in regulating the metabolic activities of cancer cells. First, GLUTs serve as the primary channels through which cancer cells absorb glucose 32. The expression of GLUT1 is significantly elevated in various cancers, making it a potential target for limiting the energy supply to cancer cells. Hexokinase (HK) serves as the initial critical enzyme in the glycolytic pathway, and its elevated activity has been linked to the malignant characteristics observed in various types of cancer 33. In particular, the overexpression of HK2 in cancer cells not only promotes glycolysis but is also linked to tumor invasiveness and chemotherapy resistance. Furthermore, 6-phosphofructo-2-kinase (PFKFB3) functions as a vital regulatory element within the glycolytic pathway by facilitating the activation of phosphofructokinase-1 (PFK-1) through the enzymatic production of fructose-2,6-bisphosphate, which in turn enhances the glycolytic process 34. Inhibiting PFKFB3 can reduce the metabolic rate of cancer cells, offering a novel strategy for cancer treatment. Furthermore, pyruvate kinase M2 (PKM2) is the final key enzyme in the glycolytic pathway. Its aberrant activation in cancer cells is closely associated with cancer proliferation and survival. PKM2 inhibitors, such as TP-1454, have demonstrated potential therapeutic effects on certain cancers in clinical trials 35.

In addition to the key targets of glucose metabolism mentioned above, the interplay between glucose metabolism and other metabolic pathways within the cancer niches should not be overlooked. For instance, lactic acid produced during glycolysis is not merely a by-product of energy metabolism, it also contributes to tumor immune evasion by modulating pH levels and influencing immune cell function 36. Consequently, the regulation of lactate metabolism, such as the inhibition of LDHA, may offer novel strategies for cancer treatment.

Cancer cells can efficiently supply energy and synthetic materials for their rapid proliferation by altering glucose metabolism. These modifications in metabolic pathways not only elucidate the underlying biological mechanisms supporting cancer cell survival and proliferation but also offer potential new targets for cancer treatment. Developing treatment approaches that focus on disrupting the distinct metabolic pathways and pivotal metabolic enzymes of cancer cells holds promise for advancing cancer therapy.

#### 2.1.2. Amino acid metabolism

**Figure 5:**
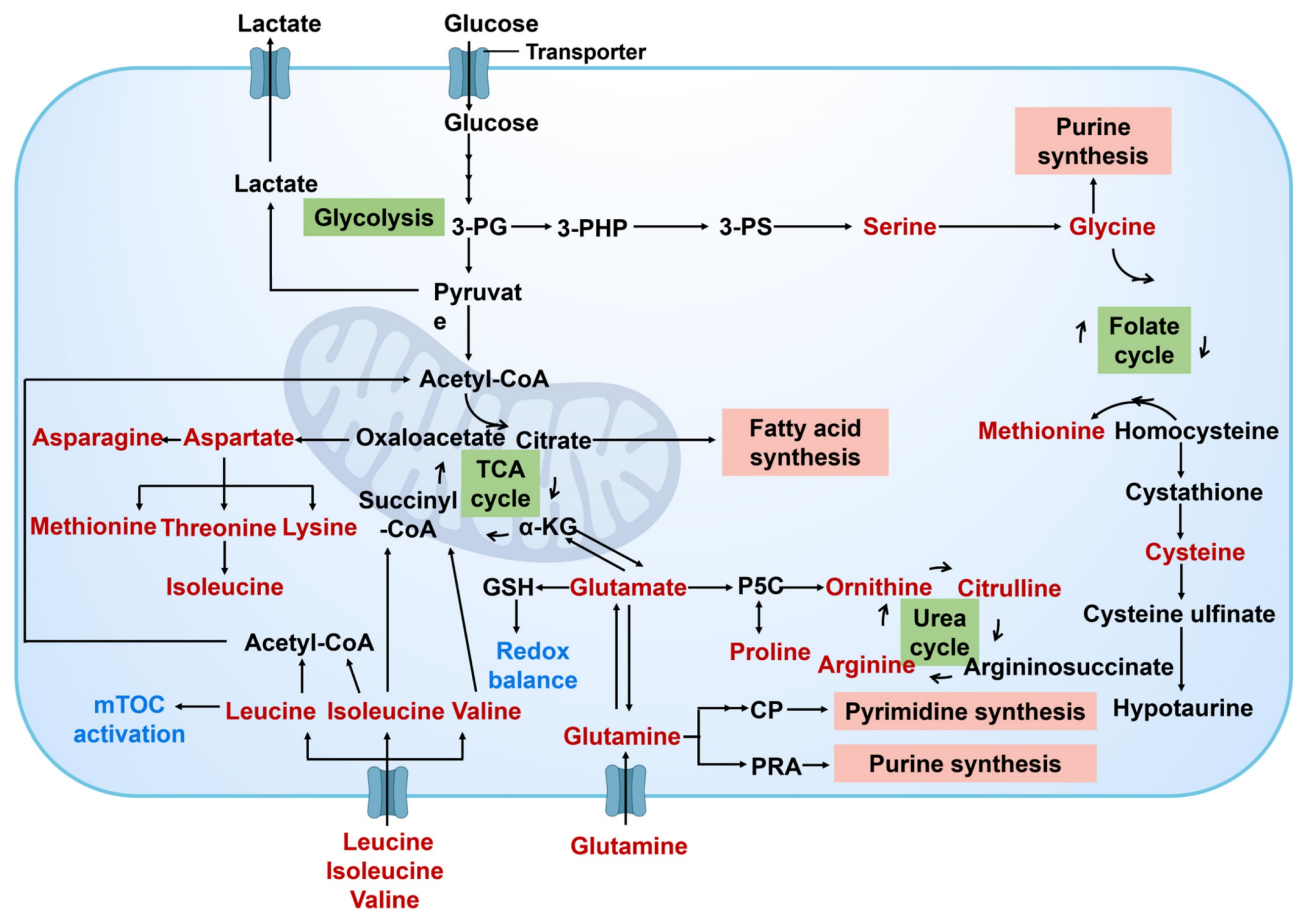
Amino acid metabolism in cancer cell. Amino acid metabolism in cancer cells and its crosstalk with other metabolism pathways. Amino acids synthesis, utilization, and involvement in other metabolism pathways are usually altered in cancer cells. 3-PG, 3-phosphoglycerate; 3-PHP, 3-phosphohydroxypyruvate; 3-PS, 3-phosphoserine; α-KG, α-ketoglutarate; GSH, glutathione; P5C, pyrroline-5-carboxylate; CP, carbamoyl phosphate; PRA, phosphoribosyl amine.

Amino acid metabolism plays a crucial role in the metabolic reprogramming of cancer cells **(Figure 5)**. This process is essential for ensuring the sufficient supply of key amino acids to facilitate protein synthesis and cell proliferation. Moreover, amino acid metabolism contributes to various cellular functions in cancer cells, including energy production, support for antioxidant defense mechanisms, and facilitation of cell signal transduction. Cancer cells modify their uptake and metabolism of specific amino acids to enhance their capacity for growth, survival, and metastasis 37. Among the various amino acids involved in this metabolic process, the glutamine metabolic pathway holds particular significance. Glutamine serves as a versatile nutrient crucial for energy synthesis, maintenance of redox homeostasis, macromolecular synthesis, and signal transduction in cancer cells. Through the action of glutaminase (GLS), glutamine is converted to glutamate, which is further metabolized into α- Ketoglutaric acid (α-KG) before entering the TCA cycle to provide energy and synthetic materials for cancer cells 38. α-KG can also undergo carboxylation into citric acid in the cytoplasm under the action of isocitrate dehydrogenase 1 (IDH1) and participate in the synthesis of fatty acids. α-KG can also be produced during the catalysis of glutamine to enhance the synthesis of non-essential amino acids. Glutamic acid, a product of glutamine metabolism, interacts directly with the proline and glutathione (GSH) biosynthesis pathway to uphold intracellular redox balance. Furthermore, glutamine participates in the synthesis of other amino acids through transamination, thereby bolstering the adaptability and viability of cancer cells 39.

Cancer cells can also enhance the metabolism of other amino acids, such as serine and glycine. These amino acids play a crucial role in protein synthesis and act as precursors for some important biological molecules like glutathione, which help to maintain the cellular redox balance and combat oxidative stress. Through the alterations in these metabolic pathways, cancer cells can efficiently utilize available nutrients, survive, and proliferate in the harsh microenvironment with inadequate nutrition supply.

Cancer cells demonstrate a significant reliance on particular amino acids, notably arginine and proline, which are essential for tumor growth, immune response regulation, and adapting to hypoxic conditions. To address the scarcity of these amino acids in the extracellular environment, cancer cells enhance the synthesis of essential amino acids by activating specific metabolic pathways. This process involves the upregulation of metabolic enzymes like prolyl hydroxylase and arginase to control amino acid concentrations, facilitating the adaptive proliferation of cancer cells.

Consequently, the metabolism of amino acids within cancer cells is integral to tumor growth and survival. These cells adapt to the demands of accelerated proliferation by increasing the uptake and metabolic processing of particular amino acids, in addition to modulating the microenvironment to facilitate their survival and spread. Within these metabolic pathways, the regulation of essential targets and signaling cascades is of paramount importance.

Aspartate metabolism plays a crucial role in the functioning of cancer cells, contributing to both cellular synthesis and energy metabolism through amino acids derived from the aspartate family 40. The increased activity of key enzymes, such as aspartate aminotransferase (AST) and glutamate dehydrogenase (GLUD), provides essential biosynthetic precursors and energy resources necessary for the survival and proliferation of cancer cells 41. The upregulation of these enzymes not only enhances metabolic flexibility in cancer cells but may also affect their responsiveness to therapeutic interventions. Furthermore, the abnormal activation of tryptophan metabolism is closely associated with tumor growth and immune evasion 42. Tryptophan metabolites, including kynurenine and indole, can modulate the functions of immune cells and foster an immunosuppressive environment within the cancer niches. Targeted inhibition of key enzymes, such as tryptophan hydroxylase (TPH) and indoleamine-2,3-dioxygenase (IDO), may aid in restoring immune surveillance and inhibiting tumor progression 43.

Arginine metabolism plays a crucial role in regulating the immune response within the cancer niches. Arginase is upregulated in various cancers, and its metabolites, such as ornithine and nitric oxide (NO), can influence the activation of immune cells and facilitate tumor immune evasion 44. Consequently, the arginine metabolic pathway and its key enzymes have emerged as potential targets for cancer immunotherapy. Similarly, methionine metabolism is also significant in cancer cells. The increased activity of key enzymes, such as methionine adenosyltransferase (MAT) and methionine synthase (MS) 45, provides essential methyl donors for cancer cells, participates in DNA and protein methylation processes, and impacts gene expression and cell signaling pathways.

The changes observed in the metabolism of amino acids within cancer cells serve to provide necessary nutrients and energy for cellular growth, while also influencing processes such as cell death, cell cycle progression, and cell signal transduction. This underscores the diverse functions of amino acids metabolism in the progression of cancer. By designing treatment strategies that target the unique amino acid metabolic pathways and key enzymes specific to cancer cells, it may offer effective ways to inhibit the progression of cancer.

#### 2.1.3. Lipid metabolism

**Figure 6:**
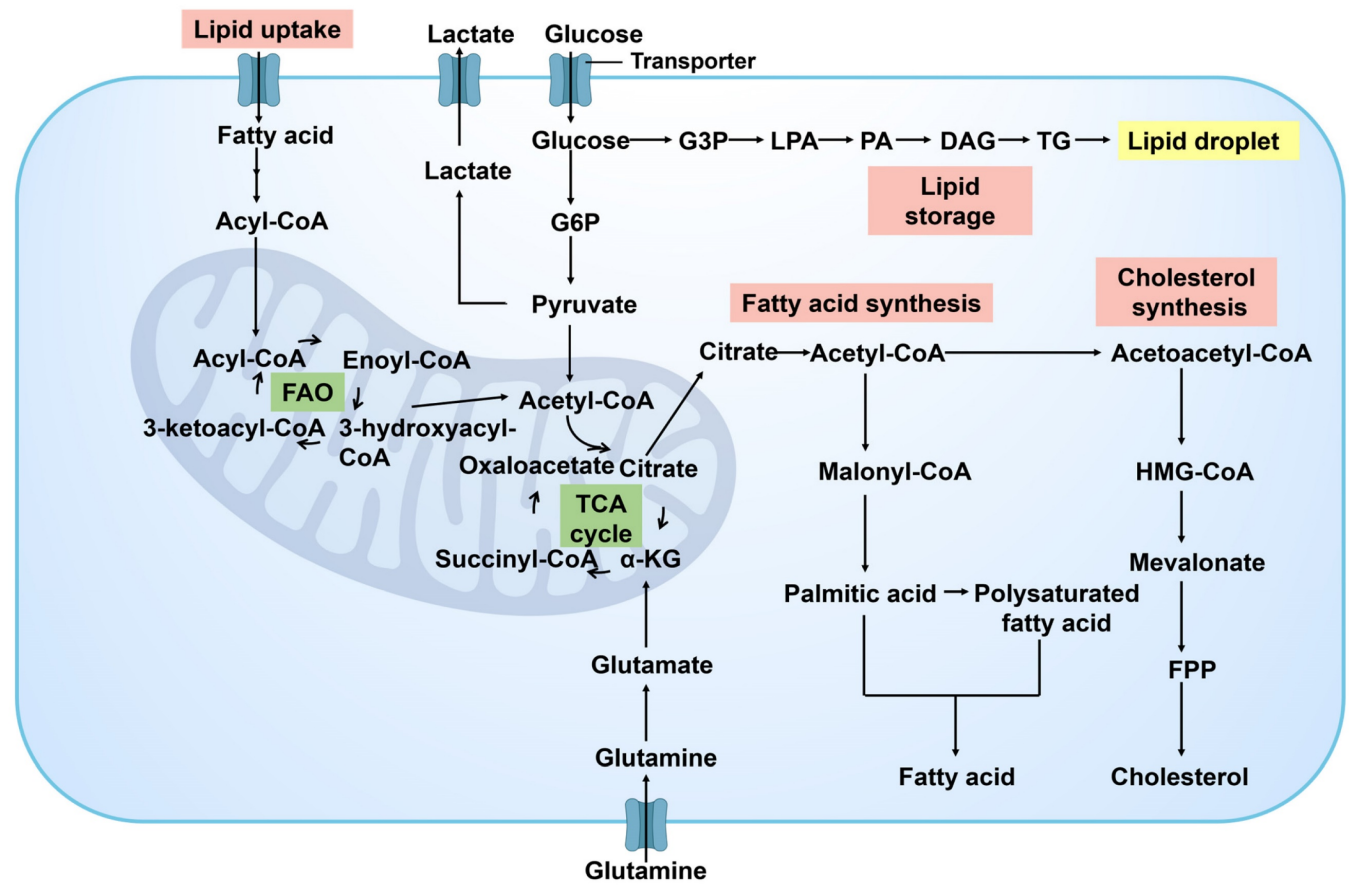
Lipid metabolism in cancer. Lipid metabolic processes include *de novo* lipogenesis, fatty acid (FA) uptake, FA oxidation (FAO), cholesterol synthesis and lipid storage. G3P, glyceraldehyde-3-phosphate; LPA, lysophosphatidic acid; PA, phosphatidic acid; DAG, diacylglycerols; TG, triglyceride; G6P, glucose 6-phosphate; HMG-CoA, 3-hydroxy-3-methylglutaryl-CoA; FPP, farnesylpyrophosphate; α-KG, α-ketoglutarate.

In the progression of cancer, the reprogramming of lipid metabolism is one of the key strategies for cancer cells to meet the demands of their proliferation and metastasis. Cancer cells modulate the synthesis, decomposition, and accumulation of lipid to ensure a continuous supply of energy and facilitate the dynamic restructuring of cell membrane. This adaptive process is vital for supporting the evolving growth requirements of cancer cells and sustaining their malignant phenotype **(Figure 6)**. As tumor progress and encounter nutrient limitations, cancer cells rely on the reprogramming of lipid metabolism to ensure their survival. By manipulating energy metabolism, cancer cells drive rapid cell growth and proliferation, ultimately contributing to cancer development. The enhancement of lipid metabolism in cancer cells not only provides energy and biosynthetic precursors but also produces important signaling molecules that play roles in regulating cell survival, proliferation, and migration 46. Cancer-related changes in lipid metabolism include increased lipogenesis, enhanced lipid uptake in the extracellular microenvironment, and improved lipid storage and mobilization of intracellular lipid droplets 47. Lipid accumulation drives cancer development by providing energy for cancer cells and serving as essential components for the synthesis of cell membrane phospholipids.

Cancer cells have a propensity to upregulate the fatty acid synthesis pathway in order to fulfill their demand for cell membrane phospholipids and other lipid molecules 48. Fatty acids (FA) are fundamental building blocks for synthesizing biofilms and serve as the primary energy source for cells. They also participate in maintaining intracellular homeostasis as cell signaling molecules. The fatty acid oxidation (FAO) pathway facilitates lipid catabolism. The formation and utilization of lipid droplets are also regulated in cancer cells. They serve as reservoirs of energy and signaling molecules, aiding cancer cells in maintaining survival during nutrient deficiency 49. Excessive free FA and cholesterol within cells are esterified into triacylglycerol (TAG) and cholesterol ester (CE) to form lipid droplets. The synthesis of lipid droplet synthesis is heightened in various tumor types, including prostate cancer, breast cancer, colorectal cancer, and liver cancer. Cancer cells also alter the fluidity and composition of their membranes by adjusting lipid metabolism pathways, impacting cell signaling and interaction with the surrounding microenvironment.

The reprogramming of lipid metabolism in cancer cells is closely associated with many tumor-specific signaling pathways, such as the PI3K/Akt and AMPK pathways. These pathways affect lipid synthesis and storage through the regulation of pivotal lipid metabolic enzymes like ATP-citrate lyase (ACLY) and fatty acid synthase (FASN) 50. Additionally, some factors in the cancer niches, such as cytokines produced by inflammatory cells, can also affect the lipid metabolism of cancer cells, thereby facilitating their proliferation and metastasis 51.

There are many key enzymes involved in lipid metabolism, such as FASN and ATP citrate lyase. Research has indicated that several enzymes and molecules linked to lipid metabolism exhibit heightened expression levels in tumor tissues. FASN, a crucial enzyme involved in FA synthesis, is notably overexpressed in various tumor tissues such as prostate cancer, colorectal cancer, ovarian cancer, and breast cancer, and is closely associated with chemotherapy resistance 52. The protein and mRNA expression levels of sterol regulatory element binding protein-1c (SREBP-1c) in human gastric cancer tissues were significantly higher compared to adjacent normal gastric tissues 53. The rate of positive expression of stearoyl CoA desaturase 1 (SCD1) in breast cancer tissues was significantly elevated in comparison to normal breast tissues 54. In addition, enzymes involved in the cholesterol biosynthesis pathway, such as HMG-CoA reductase, serve as crucial regulatory points in lipid metabolism within cancer cells. Cholesterol is not only a vital component of the cell membrane but also plays a significant role in cell signal transduction and cell cycle regulation 55. Inhibiting the activity of HMG-CoA reductase can disrupt cholesterol metabolism in cancer cells, thereby impacting their growth and survival.

The reprogramming of lipid metabolism in cancer cells plays a crucial role in enabling them to evade immune surveillance and develop resistance to therapeutic interventions. For example, changing the composition of membrane lipids can affect the interactions between cells and immune cells 56, and the production of some lipid signaling molecules is related to the development of drug resistance 57. Therefore, the reprogramming of lipid metabolism in cancer cells is a key factor in their adaptability, survival, and proliferation capabilities, and it provides new potential targets for cancer treatment. In-depth research in this field has revealed the complexity of cancer metabolic specificity and provided potential targets for the development of novel cancer treatment strategies targeting lipid metabolism pathways, demonstrating the great potential of metabolic intervention to inhibit cancer progression.

#### 2.1.4. Changes in other biosynthetic and bioenergetic pathways

In the metabolic reprogramming of cancer cells, changes in glucose, amino acid, and lipid metabolism, as well as alterations in other biosynthetic and bioenergy pathways, are pivotal in the progression of cancer. These modifications are essential for providing the necessary raw materials and energy to facilitate the synthesis of nucleotides, proteins, and other biological macromolecules during the accelerated growth of cancer cells. In particular, cancer cells regulate the nucleotide metabolic pathways to fulfill the requirements for DNA and RNA synthesis, which are essential to support their rapid cell cycle and proliferation 58. At the same time, cancer cells also alter the one-carbon metabolism pathways 59, which are crucial for preserving methylation patterns and genetic stability, thereby affecting gene expression and the epigenetic traits of tumors.

Furthermore, cancer cells reprogram their mitochondrial functions, including adjustments in oxidative phosphorylation and the electron transport chain, to adapt to energy demands and resist oxidative stress. These changes contribute to the survival of cancer cells in a hypoxic environment, and also promote the redistribution of metabolites to meet the needs of biosynthesis. Changes in enzyme activity and reactivation of metabolic pathways in cancer cells, such as the enhancement of PPP, are also part of metabolic reprogramming. This process provides cells with reducing power and ribose to support rapid proliferation.

These extensive changes in metabolic pathways illustrate the adaptive mechanisms employed by cancer cells to meet the demands for proliferation, survival, and metastasis through various metabolic reprogramming processes. These changes not only provide energy and biosynthetic precursors but also further promote the tumor development by affecting cell signal transduction, gene expression, and cell cycle regulation. In conclusion, metabolic reprogramming plays a pivotal role in regulating the progression of tumors, impacting numerous biological properties of cancer cells, and facilitating their proliferation, growth, invasion, and distant metastasis. The research on metabolic remodeling provides new ideas and methods for cancer treatment.

### 2.2. Potential mechanism of accelerating cancer progression

The mechanisms of cancer cell metabolic reprogramming are diverse in promoting growth and proliferation, and have a profound impact on tumor development. The core of this process is the Warburg effect, that is, cancer cells can rapidly generate ATP and necessary biosynthetic intermediates even under sufficient oxygen supply by enhancing the glycolysis process, so as to support rapid cell division and growth. At the same time, cancer cells enhance lipid metabolism, particularly the synthesis of fatty acids, to supply materials for the construction of new cell membranes to support their rapid proliferation requirements. In addition, by regulating the metabolism of key amino acids such as glutamine, cancer cells not only meet their own energy needs, but also provide raw materials for the synthesis of nucleotides and non-essential amino acids. Moreover, the potential mechanisms of cancer cell metabolic reprogramming involve multiple levels of complex regulation that promote cancer growth and proliferation.

#### 2.2.1. Gene mutations and epigenetic modifications

During the metabolic reprogramming of cancer cells, gene mutations and epigenetic modifications are pivotal in driving tumor progression and proliferation. Specific gene mutations such as those in tumor suppressor genes or proto-oncogenes can directly impact cellular metabolic pathways. For instance, mutations in genes like p53 60, KRAS 61, and PI3K, commonly observed in tumors, not only affect the signaling pathways of cell proliferation and apoptosis but also reconfigure the cellular metabolic network. The protein encoded by the p53 gene is a crucial regulator of cell cycle progression and DNA damage repair. Mutations in the p53 gene, prevalent in various tumors, can induce metabolic alterations like heightened glycolysis and lipid synthesis, thereby fostering cancer cell growth and proliferation. These mutations can also activate multiple signal pathways and further regulate the metabolic network to meet the needs of tumor growth. For example, the PI3K/Akt/mTOR signaling pathway 62 is instrumental in regulating cell growth, metabolism, and survival. Dysregulation of this pathway is linked to several cancers, including breast cancer and colorectal cancer. Mutation in the PIK3CA gene can lead to the excessive activation of the signaling pathway, promoting glucose uptake and glycolysis to provide the energy and biosynthetic substances necessary for cancer cell growth.

**Figure 7:**
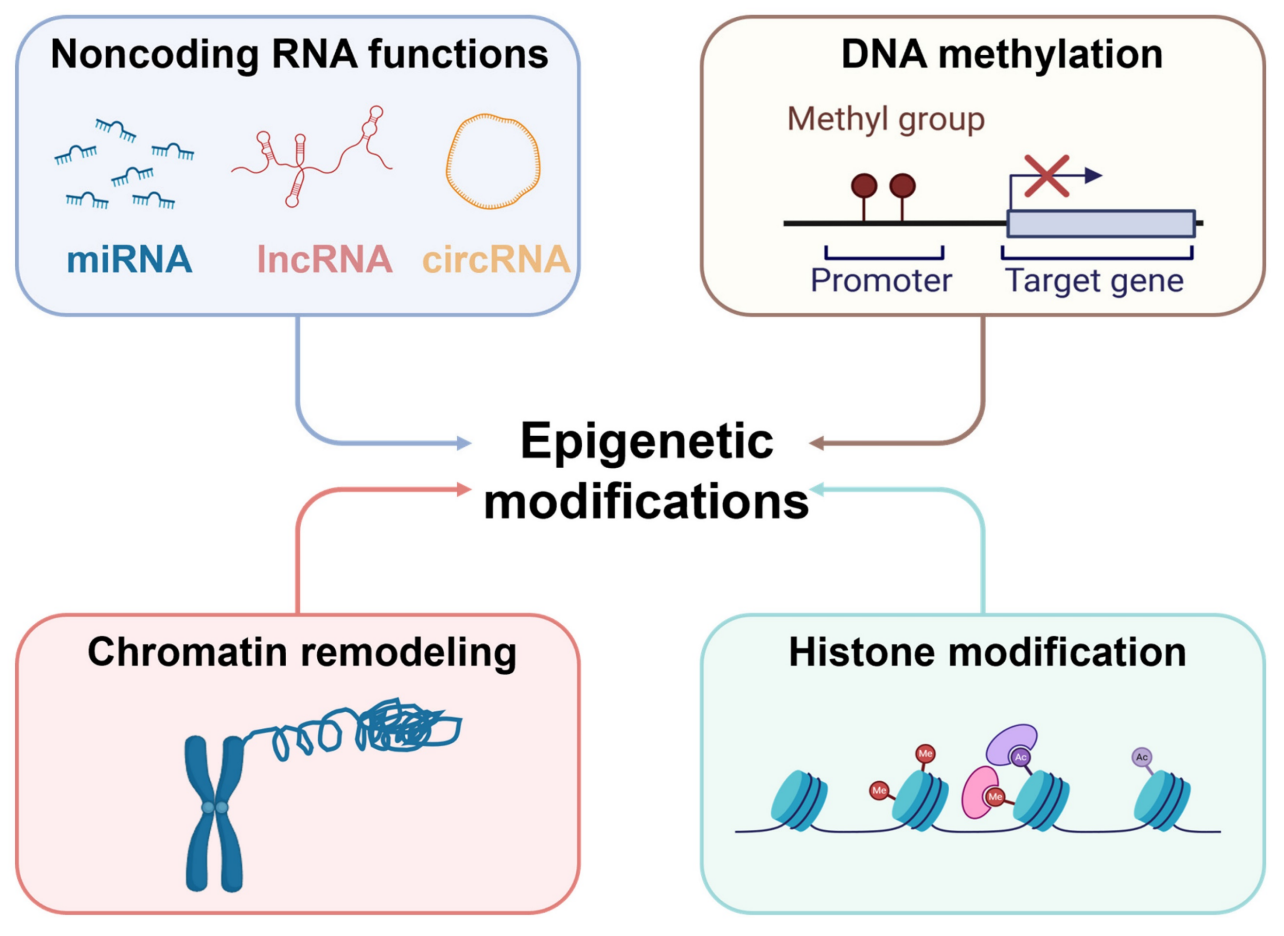
Epigenetic modifications in cancer. The mechanisms of epigenetic modification mainly include DNA methylation, histone modification, chromatin remodeling and noncoding RNA functions. Epigenetic modification mark plays an essential role in tumorigenesis and development.

Simultaneously, epigenetic modifications, including DNA methylation, histone modification, non-coding RNA regulation, and chromatin remodeling, also play a decisive role in the metabolic reprogramming of cancer cells 63-65** (Figure 7)**. Epigenetic modifications encompass heritable changes in gene expression that do not involve modifications to the DNA sequence 66. These modifications can not only regulate the expression of specific metabolic genes but also affect entire metabolic pathways and cellular states, thereby influencing the growth and proliferation of cancer cells. For instance, modulation of HIF-1α activity or regulation of c-Myc can lead to alterations in amino acid and lipid metabolism, supporting rapid cell proliferation 67.

The combination of genetic mutations and epigenetic modifications facilitates the ability of cancer cells to modulate their metabolism in order to sustain rapid proliferation, as well as to acclimate to variations in the cancer niches, such as hypoxia and malnutrition, thereby enhancing their survival and invasion capabilities. This metabolic adaptability and flexibility are key to tumor development. They also offer new strategies and targets for cancer treatment, particularly in targeted therapy for metabolic pathways and metabolic regulators. This development introduces a promising direction for the treatment of cancer.

#### 2.2.2. Influence of cancer niches

Cancer niches is a critical factor influencing the metabolic reprogramming of cancer cells, facilitating tumor growth and proliferation. The distinct characteristics of cancer niches, including hypoxia, acidic pH, and nutrient restriction, impose selective pressures on the metabolic pathways of cancer cells, necessitating adaptations to ensure their survival and proliferation. Hypoxic within the cancer niches induces cancer cells to enhance glycolysis, inhibit aerobic respiration, and stimulate neovascularization to improve oxygen supply through the activation of HIF-1α and other signaling pathways 68. In addition, hypoxia can promote the regulation of specific amino acid and lipid metabolic pathways, supporting the energy production and biosynthesis of cancer cells.

Nutrient limitations in cancer niches necessitate cancer cells to utilize alternative metabolic pathways, such as FAO and autophagy, to fulfill their energy and biosynthetic requirements. In addition, the cell-cell interactions in cancer niches also significantly impact the metabolism of cancer cells. For instance, cancer-associated fibroblasts (CAFs) 69, immune cells, and vascular endothelial cells can affect the metabolic status of cancer cells through secreted factors or direct cell contact, thereby facilitating their growth and proliferation.

Cancer cells can further shape the local environment to promote tumor growth by affecting the cancer niches, such as altering its pH levels and secreting metabolites like lactic acid. These changes may also impact the function of immune cells and facilitate tumor evasion from immune surveillance. Therefore, the interaction between cancer niches and cancer cells, along with their impact on metabolic pathways, constitute a complex network that influence tumor growth and proliferation. This network offers potential targets for intervention in cancer treatment, particularly in strategies targeting the cancer niches and metabolic adaptability. A comprehensive understanding of these interactions and mechanisms can lead to the development of novel therapeutic strategies that specifically target the metabolic reprogramming and adaptability of tumors in the niches, thereby impeding tumor progression.

#### 2.2.3. Regulation of signal transduction pathway

**Figure 8:**
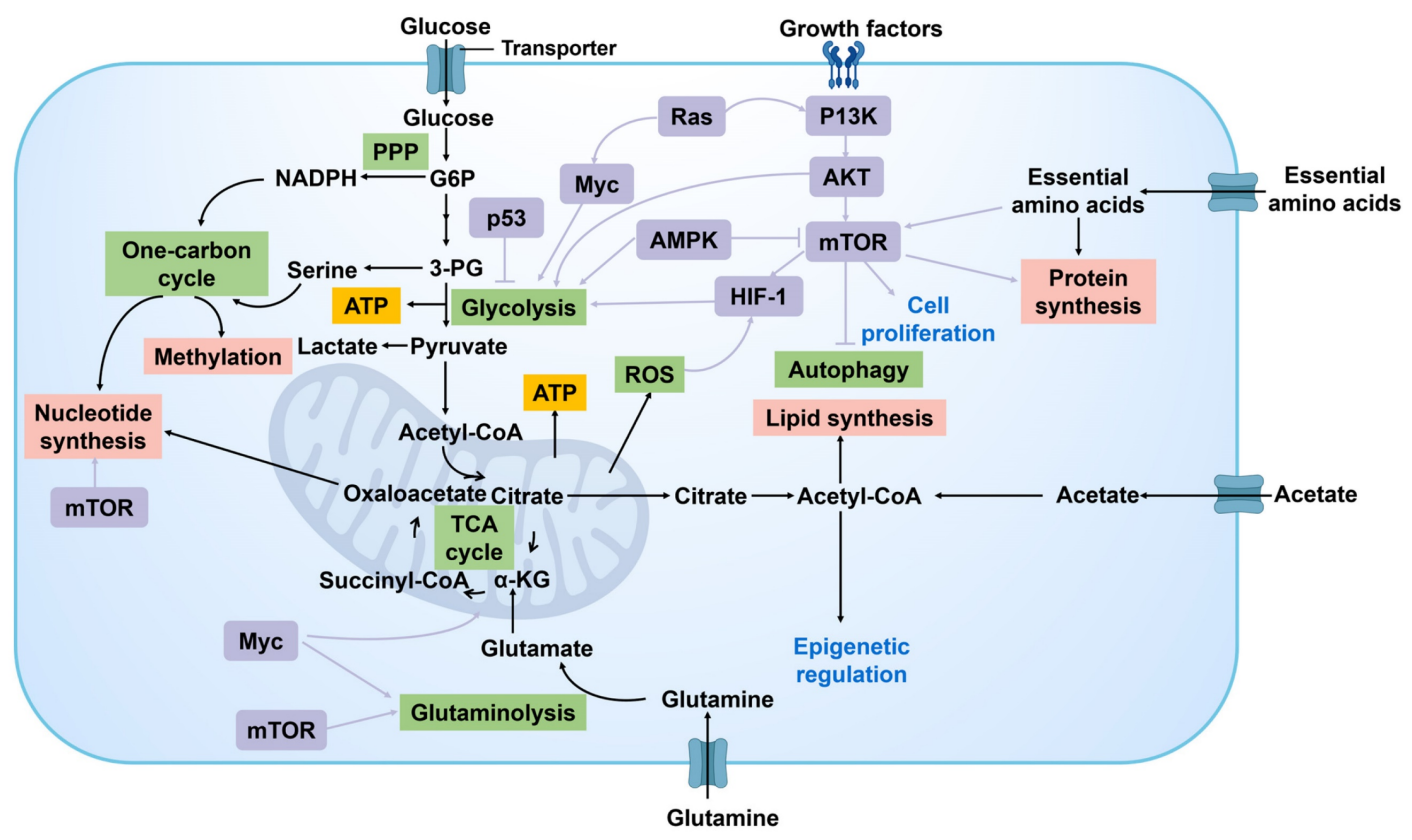
Signaling pathways regulating cancer cell metabolism. G6P, glucose 6-phosphate; 3-PG, 3-phosphoglycerate; PPP, pentose phosphate pathway; α-KG, α-ketoglutarate; ATP, adenosine triphosphate; ROS, reactive oxygen species; mTOR, mammalian target of rapamycin; P13K, phosphatidylinositol 3-kinase; AKT, protein kinase B; HIF-1, hypoxia inducible factor-1; AMPK, adenosine 5'-monophosphate (AMP)-activated protein kinase.

Metabolic reprogramming of cancer cells is a critical factor in the modulation of signal transduction pathways to promote tumor growth and proliferation** (Figure 8)**. Key signaling pathways such as PI3K/Akt, mTOR, and AMPK not only regulate cell survival and proliferation but also directly affect the metabolic status of cancer cells. For example, the activation of the PI3K/Akt pathway can enhance glycolysis, providing a rapid supply of energy and intermediates required for biosynthesis 70. The mTOR signaling pathway, being a primary regulator of cellular growth and metabolism, supports the proliferative requirements of cancer cells by amplifying protein synthesis and suppressing autophagy 71. AMPK, serving as an energy sensor, can be activated during energy deficiency to regulate the metabolic equilibrium of cancer cells for sustaining energy provision 72. In addition, HIF-1α is a key transcription factor under hypoxia that can promote the expression of a series of genes related to metabolic reprogramming. Activation of HIF-1αsignaling pathways can promote glycolysis, lactate production, and angiogenesis, enabling cancer cells to acclimate to hypoxic surroundings and bolster their growth and proliferation through heightened energy generation and nutrient transportation.

The interaction between signal transduction pathways forms a complex regulatory network. This network facilitates rapid responses of cancer cells to fluctuations in nutritional and energy conditions, while also affording them diverse mechanisms to adjust and exploit variations in the cancer niches.

In summary, these complex regulatory mechanisms not only ensure the supply of energy and synthetic raw materials for cancer cells but also facilitate their ability to adjust to variations in the microenvironment and treatment pressures, thereby promoting their growth, proliferation, and metastasis. Therefore, a comprehensive comprehension of the fundamental metabolic reprogramming mechanisms in cancer cells holds substantial importance in elucidating the biological behavior of tumors and formulating novel therapeutic targets and strategies.

## 3. Immune cell metabolism

### 3.1. Immune niches

Cancer niches is a complex environment for the survival of cancer cells 73-75. It is composed of cancer cells, stromal cells, fibroblasts, infiltrating immune cells, secreted products of the corresponding cells, and extracellular matrix. It plays a decisive role in tumor progression, immune evasion, and response to treatment. Among them, the cancer immune niches are composed of a variety of cell types **(Table 1)**, with immune cells being instrumental in shaping the inhibitory microenvironment. Immune cells include T lymphocytes, B cells, NK cells, TAMs, dendritic cells (DCs), tumor-associated neutrophils (TANs), and myeloid-derived suppressor cells (MDSCs), assume distinct roles in tumor immune surveillance. Compared with normal tissues, immune cells within cancer niches play different or even opposite functions. At the same time, the changes in the metabolic pathways of immune cells lead to dual functions: while immune cells initially demonstrate an anti-tumor effect during the early stages of tumor invasion, some transition into tumor-promoting phenotypes as the tumor progresses, fostering an immunosuppressive environment that facilitates tumor immune evasion and distant metastasis 24, 76. Cancer niches exhibit the characteristics such as ion homeostasis imbalance, partial acidity, hypoxia, increased lactic acid, decreased glucose concentration, nutritional competition, and alterations in the secretome, all of which can induce metabolic reprogramming in immune cells, thereby altering their functions. This can result in a weakened inflammatory response or enhanced inhibitory function, ultimately aiding in the immune evasion of tumors 77. Therefore, the metabolic reprogramming of immune cells is the basis of their functional transformation, and it is particularly critical for the proliferation and metastasis of cancer cells.

#### 3.1.1. The metabolism of T cells

T cells are integral components of the cancer niches, and exhibit a wide range of intricate functions. Based on different surface antigens, primary CD4^+^ T cells can differentiate into T helper cells (Th) and regulatory T cells (Treg), while primary CD8^+^ T cells can evolve into CTLs in response to various cellular and molecular stimuli within cancer niches. Memory T cells (Tm) can differentiate from primary T cells or the above-mentioned T cell subsets and have the ability to respond rapidly to specific antigens. Different subpopulations of Th cells coordinate the functions of immune effector organs such as CTLs and macrophages through the release of various cytokines. Treg cells modulate the immune response by directly inhibiting or secreting inhibitory cytokines. CTLs eliminate target cells either by releasing perforin and granzyme, or induce target cell death by expressing apoptosis-related ligands and secreting TNF-α. These complex interactions and regulatory mechanisms together constitute an important defense mechanism of the body's anti-tumor immunity.

The metabolic characteristics of T cells are fundamental to their immune function. These characteristics change dynamically with the activation, proliferation, and differentiation of T cells. In the quiescent state, T cells primarily generate energy from mitochondria via oxidative phosphorylation to fulfill their basic survival requirements. This process is more efficient, but the energy production is slower and heavily reliant on oxygen. At this time, the stimulation signal of the T cell receptor (TCR) maintains the low basal activity of the mTORC1 pathway through RasGRP and the cytokine IL-7 signaling 78. Upon encountering antigens and subsequent activation, T cells swiftly elevate glycolysis to mount a rapid immune response. Even under conditions of sufficient oxygen, T cell can quickly produce a large amount of ATP and intermediates needed for biosynthesis through the Warburg effect. This supports their rapid proliferation and functional differentiation. In addition, activated T cells also increase the metabolism of amino acids, especially glutamine, and lipid metabolism, further supporting cell proliferation, differentiation, and maintaining their effector function.

In the process of T cell differentiation, various subtypes of T cells display distinct metabolic characteristics to accommodate their specific functional requirements. For example, effector T cells, includingTh1, Th17, and cytotoxic T cells, are characterized by high levels of glycolytic activity, whereas memory T cells rely on oxidative phosphorylation and lipid oxidation to maintain their long-term survival and rapid response to specific antigens again. Tregs perform their immunosuppressive functions by regulating their own metabolism as well as those of other immune cells. The regulation of T cell metabolism is not only related to energy production but also involves cell signal transduction, epigenetic modification, and cell fate determination, all of which are indispensable parts of their immune function. Therefore, a comprehensive understanding of the metabolic characteristics of T cells is crucial for uncovering their immune regulation mechanism and developing innovative immunotherapeutic strategies.

#### 3.1.2. The metabolism of macrophages

Macrophages, essential elements of the cancer niches, exhibit significant plasticity and diversity in their metabolic characteristics across different activation and functional states. In the quiescent state, macrophages predominantly rely on oxidative phosphorylation for energy production to sustain energy equilibrium and execute fundamental phagocytic functions. Upon encountering pathogens or inflammatory signals, macrophages can be activated and differentiated into two main phenotypes: pro-inflammatory M1 and anti-inflammatory M2, which exhibit significant differences in metabolism 79.

M1 macrophages enhance the glycolysis process to expedite energy provision, facilitating the synthesis of substantial of pro-inflammatory factors, reactive oxygen species, and nitrogen intermediates to combat invading microorganisms and participate in inflammatory responses. In addition, M1 macrophages also increase their glycolytic activity by inhibiting the oxidative phosphorylation of pyruvate within mitochondria, thereby enhancing their pro-inflammatory and bactericidal abilities 80. Conversely, M2 macrophages predominantly rely on oxidative phosphorylation and FAO for energy production. This metabolic pathway enables them to carry out functions such as tissue regeneration, clearance of apoptotic cells, and regulation of the immune response. This metabolic mode of M2 macrophages is more efficient and durable, which is suitable for their needs in the process of chronic inflammation and tissue repair.

The adaptability of macrophages in their metabolic processes not only demonstrates their versatility in immune responses but also underscores the significance of metabolic regulation in facilitating macrophage functions. This metabolic plasticity enables macrophages to effectively execute their immune function under various physiological and pathological conditions.

Tumor-associated macrophages (TAMs) are essential cell types within the cancer niches. They undergo metabolic reprogramming to adapt to the cancer niches, acquiring immunosuppressive and tumor-promoting functions in the process. TAMs primarily rely on glycolysis and fatty acid oxidation to meet their energy demands, which is closely associated with tumor immunosuppression and the promotion of tumor growth 81. TAMs contribute to tumor immune evasion and metastasis through various metabolic pathways. For instance, TAMs utilize glucose metabolism to regulate epigenetic modifications, signal transduction, O-glycosylation, and other post-translational modifications of numerous proteins, thereby facilitating tumor polarization and function. Furthermore, glucose metabolism can initiate the exchange of metabolites, cytokines, and signaling molecules between TAMs and other cells in cancer niches, ultimately reshaping the cancer niches.

Metabolic reprogramming of TAMs is closely linked to tumor metastasis and drug resistance. TAMs enhance their tumor-promoting functions through the hexosamine biosynthesis pathway (HBP) 82. Furthermore, the glucose metabolism of TAMs modifies cathepsin B via O-linked-β-N-acetylglucosamine (O-GlcNAcylation), a process facilitated by O-linked N-acetylglucosamine transferase (OGT) in the lysosome. This modification promotes tumor metastasis and resistance to chemotherapy. These findings reveal a novel mechanism by which TAMs contribute to tumor immune metabolism and immune evasion.

The metabolic changes in TAMs not only influence their own functions but also sustain and enhance the metabolic phenotype of tumors through interactions with other cells in the cancer niches. For instance, TAMs compete with tumor cells for nutrients, particularly glucose. This competitive dynamic alters the glucose metabolic pathway in TAMs, leading them to exhibit metabolic characteristics similar to those of tumor cells 83. Furthermore, TAMs promote tumor angiogenesis and immunosuppression by secreting various cytokines and angiogenic factors, such as vascular endothelial growth factor A (VEGF-A) and adrenomedullin (AMD). This secretion facilitates tumor evasion of immune surveillance and metastasis 84.

In the cancer niches, a significant relationship exists between the metabolic heterogeneity of TAMs and tumor prognosis. For example, the subgroup of TAMs characterized by high levels of MHC II (MHC II^hi^) is associated with the inhibition of the TCA cycle, whereas the subgroup with low levels of MHC II (MHC II^lo^) is linked to enhanced oxidative and glycolytic metabolism 85, 86. Furthermore, lactic acid has been shown to augment the oxidative metabolism of MHC II^lo^ TAMs, facilitate L-arginine metabolism, and enhance their capacity to suppress T cell activity 87. TAMs exhibit a range of functions within the cancer niches, influenced by their metabolic profiles, which include promoting tumor growth, angiogenesis, invasion, metastasis, and the suppression of anti-tumor immune responses. Consequently, targeting the metabolic pathways of TAMs may represent a promising therapeutic approach for cancer treatment.

#### 3.1.3. The metabolism of B cells

B cells play a crucial role in the immune system, and their metabolic properties are essential for their development, differentiation, antibody production, and other immune functions. In the quiescent state, B cells, like other immune cells, primarily depend on oxidative phosphorylation for energy metabolism and sustain a low metabolic rate to support fundamental cellular functions. Once stimulated by specific antigens and activated with the assistance of T cells, B cells will enhance glycolysis to rapidly provide energy and necessary biosynthetic precursors to support their proliferation and differentiation into plasma cells secreting antibodies 88. In addition, during the differentiation process, B cells also boost their utilization of amino acids and lipids to supply additional energy and synthetic materials for cell proliferation and antibody production.

The regulation of metabolic pathways is particularly important in the development and differentiation of B cells. For example, the proliferation and affinity maturation of B cells in the germinal center reaction require a significant amount of energy and biosynthesis. At this time, the obvious enhancement of glycolysis and amino acid metabolism is observed 89. The formation of memory B cells and long-lived plasma cells requires distinct metabolic assistance to maintain their long-term survival and function.

Therefore, the metabolic regulation of B cells is crucial for their immune function, encompassing energy production, cell proliferation, differentiation, and antibody production. These metabolic characteristics not only reflect the adaptability of B cells to energy and biosynthetic demands under different functional states but also offer an important perspective for a comprehensive understanding of the role of B cells in the immune response. Furthermore, they provide a potential strategy for immune regulation of B cell metabolism.

#### 3.1.4. The metabolism of dendritic cells

DCs are recognized as the most potent professional antigen-presenting cells, playing a crucial role in the maintenance and regulation of immune responses 90. In a quiescent state, DCs predominantly utilize oxidative phosphorylation (OXPHOS) within mitochondria for energy production. However, upon activation by toll-like receptor (TLR) agonists or lipopolysaccharide (LPS), there is a notable shift in the metabolic profile of DCs towards heightened glycolysis 91. This shift involves the rapid degradation of glycogen stored in DCs through glycolysis to promptly generate energy, leading to the conversion of the metabolic pathway controlled by OXPHOS to aerobic glycolysis. This metabolic reprogramming allows DCs to efficiently generate a substantial amount of ATP to fulfill their functional requirements, such as antigen processing and T cell activation.

The metabolic reprogramming of DCs is regulated by various signaling molecules, including HIF-1α, PI3K, and Akt, which are crucial in influencing the metabolic pathways and metabolite production of DCs, thereby regulating their maturation, differentiation, and migration 92. Some specific metabolic enzymes such as succinate-CoA ligase β subunit Suclg2 also play a significant role in the metabolism and immune regulation of DCs 93. In addition, the metabolic profile of DCs is closely related to their behavior within cancer niches. In tumor-infiltrating DCs, glycolysis and stimulator of interferon genes (STING) signaling pathway interact in a positive feedback regulation mechanism to enhance the anti-tumor immune function of DCs 94. This heightened metabolic activity in the cancer niches allows for rapid ATP synthesize, facilitates the activation of the STING signaling pathway, and boosts the anti-tumor capabilities of DCs. This finding provides a new strategy for enhancing antitumor immunotherapy by targeting DCs metabolism.

The metabolic characteristics of DCs form the basis of their immune function. Modulating the metabolic pathways and metabolites of DCs can affect their maturation, differentiation, and migration, thereby influencing their immune function. This is of great significance for further understanding the mechanism of DCs in immune response and developing new immunotherapy strategies.

Various immune cells within the immune system have unique metabolic characteristics that enable them to play a key role in specific immune responses. Immune cells demonstrate notable variations in metabolism compared to normal tissues. The metabolic activities of normal tissues primarily focus on maintaining the basic functions and physiological needs of cells, whereas the metabolism of immune cells is highly specialized to support their specific immune functions. For instance, immune cells in a quiescent state may adopt a more conservative metabolism. Once stimulated, they promptly adjust their metabolic processes to fulfill the significantly heightened energy and biosynthetic requirements. This rapid metabolic reprogramming is the key for immune cells to perform powerful immune functions in a short period.

In general, various types of immune cells adjust their metabolic characteristics to meet their specific immune function requirements. These metabolic characteristics not only reflect the activation status and functional requirements of immune cells but also offer potential targets for developing therapeutic strategies for specific immune cell types.

### 3.2. Key metabolic targets in immune cell metabolism

The metabolism and functional regulation of immune cells is a complex process that involves multiple signaling pathways and key targets. These pathways and targets play a crucial role in maintaining immune homeostasis, promoting immune responses, and regulating the activity of immune cells in response to pathogens and tumors.

The JAK/STAT signaling pathway is a critical regulatory network 95 that responds to cytokine stimulation and involves various cytokine receptors, including IL-6, IL-10, and IFN-γ. Upon activation of these receptors, the STAT protein is activated through the autophosphorylation and transphosphorylation of JAK protein tyrosine kinases 96. Activated STAT proteins form dimers, translocate to the nucleus, and regulate the transcription of target genes involved in the growth, differentiation, and survival of immune cells, as well as the immune response to pathogens 97. The activation of STAT proteins also includes negative feedback mechanisms to maintain the balance of the immune response. In immune cells, the activation of the JAK/STAT signaling pathway is essential for B cell differentiation, plasma cell formation, and the regulation of the acute phase response.

Furthermore, the NF-κB signaling pathway plays a crucial role in regulating immune and inflammatory responses 98. This pathway is involved in the regulation of various immune cytokines, including tumor necrosis factor (TNF) and interleukins (ILs), which are central to promoting inflammatory responses and activating immune cells 99. The activation of NF-κB can induce the expression of inflammation-related genes and enhance the effective functioning of immune cells. Conversely, abnormal activation of NF-κB is associated with the development of several autoimmune diseases and tumors.

In addition, the TCR signaling network plays a crucial role in T cell activation and function. The activation of the TCR triggers a series of downstream signaling events, including the phosphorylation of Zap70, the activation of PI3K, and the subsequent activation of the Akt and mTOR signaling pathways 100. These pathways collectively regulate the metabolic reprogramming of T cells, promote their clonal expansion and differentiation, and enhance their cytotoxic ability against tumor cells. In particular, the mTOR signaling pathway is essential for regulating the metabolic state of T cells, facilitating the differentiation of effector T cells, and maintaining the homeostasis of memory T cells 101.

Metabolic reprogramming in B cells is essential for the processes of antibody production and antigen presentation. Upon activation, B cells meet the energy requirements necessary for their proliferation and differentiation by enhancing metabolic pathways, including glycolysis and oxidative phosphorylation 102. Furthermore, the mTOR signaling pathway is instrumental in modulating the metabolic state of B cells, thereby supporting antibody class switching and the development of memory B cells. Furthermore, DCs are specialized cells responsible for presenting antigens. Their metabolic state significantly affects their ability to capture, process, and present antigens 103. DCs can improve their immune functionality through the regulation of glycolysis and amino acid metabolism. In particular, the mTOR signaling pathway is essential for the development, differentiation, and antigen-presenting capabilities of DCs.

In general, the metabolism and functional regulation of immune cells constitute a complex network that involves the interaction of various signaling pathways and key targets. Understanding the roles of these signaling pathways and targets in immune cell metabolism is crucial for the development of new immunotherapy strategies.

### 3.3. Immune activity and energy supply

The close relationship between immune activity and energy supply is fundamental for the maintenance of optimal immune responses and the ability to combat immune threats. The metabolic state of immune cells has a decisive impact on their functional capabilities, as these cells necessitate substantial energy and resources to execute their tasks effectively. This relationship is particularly complex in the cancer niches because cancer cells regulate the metabolism of immune cells through various mechanisms to evade immune surveillance and elimination.

Immune cells require a significant amount of energy and need to synthesize new biomolecules to execute their functions, such as pathogen or cancer cell recognition and elimination, as well as the secretion of immune regulatory factors. The primary source of energy for these cells is ATP, with its production closely related to cellular metabolic status, particularly through processes like glycolysis, oxidative phosphorylation, fatty acids β-oxidation, and other metabolic pathways. Immune cells predominantly rely on the OΧPHOS to efficiently produce energy in the quiescent state, activation prompts an increase in glycolysis to swiftly provide energy and raw materials for synthesis, even in the presence of ample oxygen. Furthermore, immune cells also need to regulate the metabolism of amino acids and lipids to support cell proliferation, differentiation, and the synthesis of effector molecules required in the process of immune response process. Consequently, the metabolic activity of immune cells plays a pivotal role in shaping their immune function.

Immune cells require a variety of nutrients in addition to energy, with glucose, amino acids, and fatty acids being crucial components. Glucose is the primary source of rapid energy supply, particularly during phases of cell activation and rapid proliferation. Amino acids serve as the fundamental building blocks for protein synthesis and contribute to various metabolic pathways that support the proliferation and function of immune cells. Fatty acids serve as a source of long-term energy storage and are involved in the formation of cell membranes and signaling molecules. Therefore, the supply of nutrients directly impacts the activity and function of immune cells 104, particularly in special conditions such as cancer niches. The high metabolic rate of cancer cells often results in nutrient depletion and inadequate oxygen supply, which can hinder the metabolic activity and immune function of immune cells while facilitating immune evasion by cancer cells. Therefore, exploring the relationship between immune activity and energy supply not only aids in understanding the functional regulation mechanism of immune cells in different physiological and pathological states but also provides an important theoretical basis for the development of new immunotherapy strategies for the metabolic regulation of immune cells.

### 3.4. Regulatory mechanism of immune cell metabolism in cancer niches

In the cancer niches, the metabolic regulation mechanism of immune cells is particularly complex due to the challenging conditions it presents, such as nutrients limitation, hypoxia, and the accumulation of metabolic waste products. These factors collaborate to influence immune cells, compelling them to modify their metabolic strategies to accommodate the cancer niches and sustain their anti-tumor function.

#### 3.4.1. Nutrient restriction

In the cancer niches, the regulatory mechanism of immune cell metabolism is influenced by various factors, with nutrients restriction of being a key factor. The scarcity of nutrients within the cancer niches is primarily attributed to the rapid proliferation of cancer cells and their high demand for nutrients. This scarcity directly hampers the metabolic processes and functional efficacy of immune cells. The absence of essential nutrients such as glucose, amino acids, and fatty acids necessitates immune cells to modify their metabolic pathways. This adjustment may involve transitioning from relying on glycolysis to other energy substances to fulfill basic energy requirements. However, this metabolic transition often results in diminished cellular functionality and a weakened immune response. Furthermore, the restriction of nutrients also affects the proliferation, differentiation, and effector function of immune cells. For example, some specific nutrients are indispensable for the normal division and activation of immune cells. The absence of these substances significantly impedes the proper development and immune function of immune cells. In addition, the restriction of nutrients may also lead to autophagy of immune cells. Autophagy is a cell self-protection mechanism that maintains the survival and function of cells by degrading and reusing intracellular components 105. However, long-term undernutrition may also lead to apoptosis or functional failure of immune cells, thereby reducing the body's immune surveillance and elimination capabilities against cancer cells. Therefore, the restriction of nutrients is one of the important factors in the metabolic regulation of immune cells in the cancer niches. It not only directly affects the metabolic state of immune cells but also indirectly influences the body's the anti-tumor immune response. In order to maintain the normal function and anti-tumor activity of immune cells, it is necessary to ensure an adequate supply of nutrients or optimize the metabolic environment of immune cells through nutritional supplementation and metabolic intervention.

#### 3.4.2. Hypoxic conditions

In the cancer niches, hypoxia play a significant role in influencing the metabolism and functionality of immune cells, thereby serving as a critical determinant in modulating immune cells activities 106. The inadequate blood supply within tumors, attributed to the rapid proliferation of cancer cells and the constriction of peripheral blood vessels, results in the establishment of a hypoxic milieu. This hypoxic state poses a formidable challenge to the immune cells infiltrating the tumor tissue. Primarily, hypoxia can damage mitochondrial function of immune cells, subsequently affecting aerobic respiration and prompting a shift towards increased reliance on anaerobic glycolysis for energy production. Nonetheless, the energy yield from anaerobic glycolysis is notably lower compared to aerobic respiration. While this metabolic transition can temporarily sustain the survival and function of cells, long-term hypoxia can diminish the metabolic efficiency and cellular functional performance. Additionally, hypoxia can also regulate the function of immune cells by influencing gene expression and signaling pathways. For example, hypoxia can induce the expression of a series of hypoxia-inducible factors (HIFs) in immune cells, which can regulate genes related to cell survival, proliferation, and migration. Notably, HIF-1α can upregulate the expression of PD-L1, a factor implicated in immune evasion within the cancer niches. Previous studies have shown that targeting HIF-1α can counteract PD-L1-mediated immune escape in the cancer niches, thereby enhancing immune surveillance in normal tissues 107. Furthermore, hypoxia may also affect the expression and function of receptors on the surface of immune cells, thereby altering their ability to recognize and eliminate cancer cells. Moreover, hypoxia may also induce apoptosis or autophagy of immune cells, further weakening the body's anti-tumor immune response. However, some immune cells can also develop adaptive mechanisms under hypoxic conditions, such as upregulating the expression of antioxidant genes to reduce the damage caused by hypoxia.

Hypoxia has a significant impact on the regulatory mechanism of immune cell metabolism in the cancer niches. It not only changes the energy metabolism of immune cells but also may affect their activation and immune function. Consequently, a comprehensive examination of the effects of hypoxia on immune cells is imperative in the context of investigating tumor immunotherapy.

#### 3.4.3. Accumulation of metabolic waste

In the cancer niches, the accumulation of metabolic byproducts plays a substantial role in influencing the regulatory mechanism of immune cell metabolism. With the continuous proliferation of cancer cells and the intensification of metabolic activities, a large number of metabolic wastes, such as lactic acid, ammonia, and other metabolites, gradually accumulate in the cancer niches. The accumulation of these wastes not only alters the pH value of the cancer niches but also may affect the function and metabolism of immune cells, either directly or indirectly.

Initially, the accumulation of acid metabolites, such as lactic acid, results in the acidification of cancer niches, leading to detrimental impacts the activity and functionality of immune cells. An acidic environment can inhibit the proliferation, activation, and cytokine production of immune cells, thereby weakening their ability to recognize and kill cancer cells 108. In addition, an acidic environment may also induce apoptosis of immune cells, further reducing the body's anti-tumor immune response. Secondly, the accumulation of ammonia and other metabolic byproducts may also have direct toxic effects on immune cells. Ammonia, a byproducts of protein metabolism 109, may elevate in the cancer niches due to disruptions in cell metabolism and the increased protein decomposition. A high concentration of ammonia will not only affect the normal metabolism of immune cells but also may cause damage to cell function. At the same time, ammonia may also affect the signal transduction pathways and gene expression patterns in immune cells, thereby altering the reactivity and effector function of immune cells. In addition to the aforementioned direct effects, the accumulation of metabolic waste may also indirectly regulate the metabolism and function of immune cells. For example, waste products like lactic acid and ammonia can alter the behavior and function of other cell types in the cancer niches, affecting the permeability of vascular endothelial cells and trigger fibroblast activation. These changes may affect the infiltration and activity of immune cells.

In addition to lactic acid and ammonia, various metabolic byproducts, such as reactive oxygen species and free radicals, may accumulate in the cancer niches. The production and accumulation of these byproducts may cause damage to immune cells and disrupt their normal metabolism and function.

In summary, the accumulation of metabolic byproducts in the cancer niches has detrimental effects on the metabolism and function of immune cells through multiple mechanisms. This accumulation may affect the immune cells response by altering the pH value of the surrounding milieu, causing toxic effects, or disrupting with the normal metabolism and function of immune cells. Therefore, in the study of tumor immunotherapy, it is necessary to consider how to reduce or eliminate these metabolic wastes to restore the normal function and activity of immune cells.

## 4. Interaction between cancer cells and immune cells

The interaction between cancer cells and the immune system constitutes a complex biological network that plays a crucial role in both tumor development and progression, as well as influencing the efficacy of immunotherapy. The immune system defends against tumors by identifying and eliminating abnormal cells, but cancer cells develop multiple mechanisms to evade immune surveillance and attack.

### 4.1. Immune recognition

Immune recognition plays a central role in the interaction between cancer cells and the immune cells, serving as the initial phase of the immune response against cancer. This process involves the recognition of specific antigens on the surface of cancer cells by immune cells. These antigens can either be neoantigens generated by tumor mutation or antigens that are typically expressed at minimal levels but are overexpressed in cancer cells. Antigen-presenting cells (APCs), such as DCs, capture and process these antigens, presenting their fragments to T cells via major histocompatibility complex (MHC) molecules 110. T cells specifically bind to the MHC-antigen complex through the TCR on their surface, leading to T cells activation and initiation of a series of immune responses against the tumor 111. CD8^+^ T cells can directly eliminate cancer cells expressing tumor antigen, while CD4^+^ T cells coordinate the entire immune response by secreting cytokines. These cytokines stimulate the production of antibodies by B cells and promote the function of CD8^+^ T cells. Moreover, immune recognition also involves the formation of immune memory, enabling a faster and more effective immune response to the same tumor antigen upon subsequent encounters. Furthermore, the effectiveness of immune recognition can be compromised by evasion mechanisms employed by cancer cells. Cancer cells can interfere with the immune recognition process through various strategies to evade the immune system's attack. Consequently, immune recognition not only serves as the foundation for the immune system to identify and combat cancer cells but also acts as a critical link for cancer cells to evade immune surveillance. Therefore, a comprehensive exploration of the molecular mechanisms and regulatory pathways of immune recognition is of vital significance for the development of more effective cancer immunotherapy strategies.

### 4.2. Immune escape

Immune escape refers to a series of strategies employed by cancer cells to evade detection and attacked by the immune system, enabling their uncontrolled proliferation. This phenomenon plays a crucial role in the dynamic interplay between cancer cells and the immune system.

In order to survive and proliferate in the host, cancer cells employ diverse mechanisms to evade or suppress immune system 112. These mechanisms include downregulating the expression of tumor antigens and MHC molecules to reduce immune cell recognition. Moreover, the upregulation of immune checkpoint molecules like PD-L1 can impede the activation and attack of T cells by binding with PD-1 on T cells and transmitting inhibitory signals 113, 114. Cancer cells also release immunosuppressive factors, such as TGF-β and IL-10, to directly inhibit the activity of immune cells or alter the immune microenvironment to promote immune tolerance. Furthermore, cancer cells recruit and activate immunosuppressive cells like Treg cells and TAMs to further establish an immunosuppressive microenvironment. In addition, cancer cells can manipulate metabolic pathways to disrupt the metabolic balance of T cells, thereby inhibiting their function. These immune escape strategies not only help cancer cells evade immune surveillance and clearance but also create favorable conditions for cancer growth, invasion, and metastasis. An in-depth understanding of the immune escape mechanism is essential for the development of more effective immunotherapeutic strategies. This includes designing methods to block the immunosuppressive pathway, enhance the recognition and killing abilities of immune cells, and restore immune cell function by interfering with cancer metabolism.

### 4.3. Metabolic crosstalk and its impact between cancer cells and immune cells

In the cancer niches, the metabolic crosstalk between cancer cells and immune cells represents a complex, bidirectional regulatory process that involves multiple precisely tuned molecular targets and signaling pathways 115. These pathways not only regulate the metabolism and function of immune cells but also influence the metabolic adaptability of tumor cells, thereby collectively impacting tumor development and the response to treatment.

The PI3K/Akt/mTOR signaling pathway plays a dual role in both cancer cells and immune cells. In immune cells, particularly T cells, the activation of this pathway can induce metabolic reprogramming, promote glycolysis and glutamine catabolism, and subsequently enhance the activation, proliferation, and effector functions of T cells, which are essential for stimulating an effective immune response 116. Conversely, in cancer cells, the continuous activation of the PI3K/Akt/mTOR pathway serves as a key driving force for the metabolic adaptability of these cells, contributing to their enhanced survival, proliferation, and invasive capabilities 117. This persistent activation may also facilitate immune escape by modulating the function of immune cells. Inhibitors targeting this pathway, such as Everolimus and Temsirolimus, have demonstrated potential in the treatment of various tumors.

As a sensitive monitor of intracellular energy states, the AMPK signaling pathway can respond rapidly to small changes in energy supply 118. In immune cells, the activation of AMPK promotes the fatty acid oxidation process, provides essential energy support, and simultaneously inhibits the mTOR pathway 119. This inhibition may further enhance the anti-tumor function of immune cells by reducing metabolic demand and improving energy utilization efficiency. In tumor cells, the activation of AMPK may limit proliferation by regulating cell metabolism and maintaining energy balance 120. AMPK activators, such as metformin, have been studied for their potential to enhance the sensitivity of tumors to immunotherapy.

The JAK/STAT signaling pathway plays a central role in regulating the differentiation, activation, and immune response of immune cells. In the cancer niches, abnormal activation of this pathway can lead to immune cell dysfunction, such as promoting the generation and survival of Tregs, thereby contributing to tumor immune escape 121. Ruxolitinib, tofacitinib, and other inhibitors targeting the JAK/STAT pathway have demonstrated efficacy in the treatment of immune-related diseases and are being explored for their application in tumor immunotherapy 122. These inhibitors aim to restore immune cell function and enhance the immune response to tumors by inhibiting the JAK/STAT pathway.

The NF-κB signaling pathway plays a crucial role in regulating immune and inflammatory responses. In cancer cells, the activation of NF-κB can promote metabolic reprogramming, enhance cell viability, and contribute to the inflammatory microenvironment of cancers, thereby facilitating cancer progression and immune evasion 123. In immune cells, NF-κB activation can stimulate the production of inflammatory factors and influence the activation and function of these cells 124. Currently, inhibitors targeting the NF-κB pathway, such as bortezomib and dexamethasone, have been utilized in various cancer treatments and have demonstrated some therapeutic efficacy 125. However, the application of these inhibitors in tumor immunotherapy requires further investigation and optimization.

The interaction of these signaling pathways forms a complex metabolic regulatory network within the cancer niches, influencing cancer development and treatment response. Understanding the regulatory mechanisms of these pathways will facilitate the development of new treatment strategies, such as combining metabolic inhibitors with immunomodulators, to enhance the efficacy of tumor therapies. Future research should further investigate the specific roles of these pathways across various cancer types and immune environments to create more effective, individualized treatment strategies. By targeting and regulating these key pathways, it is possible to remodel cancer metabolism, restore immune cell function, and improve the overall effectiveness of tumor therapies.

### 4.4. Cancer immunoediting

**Figure 9:**
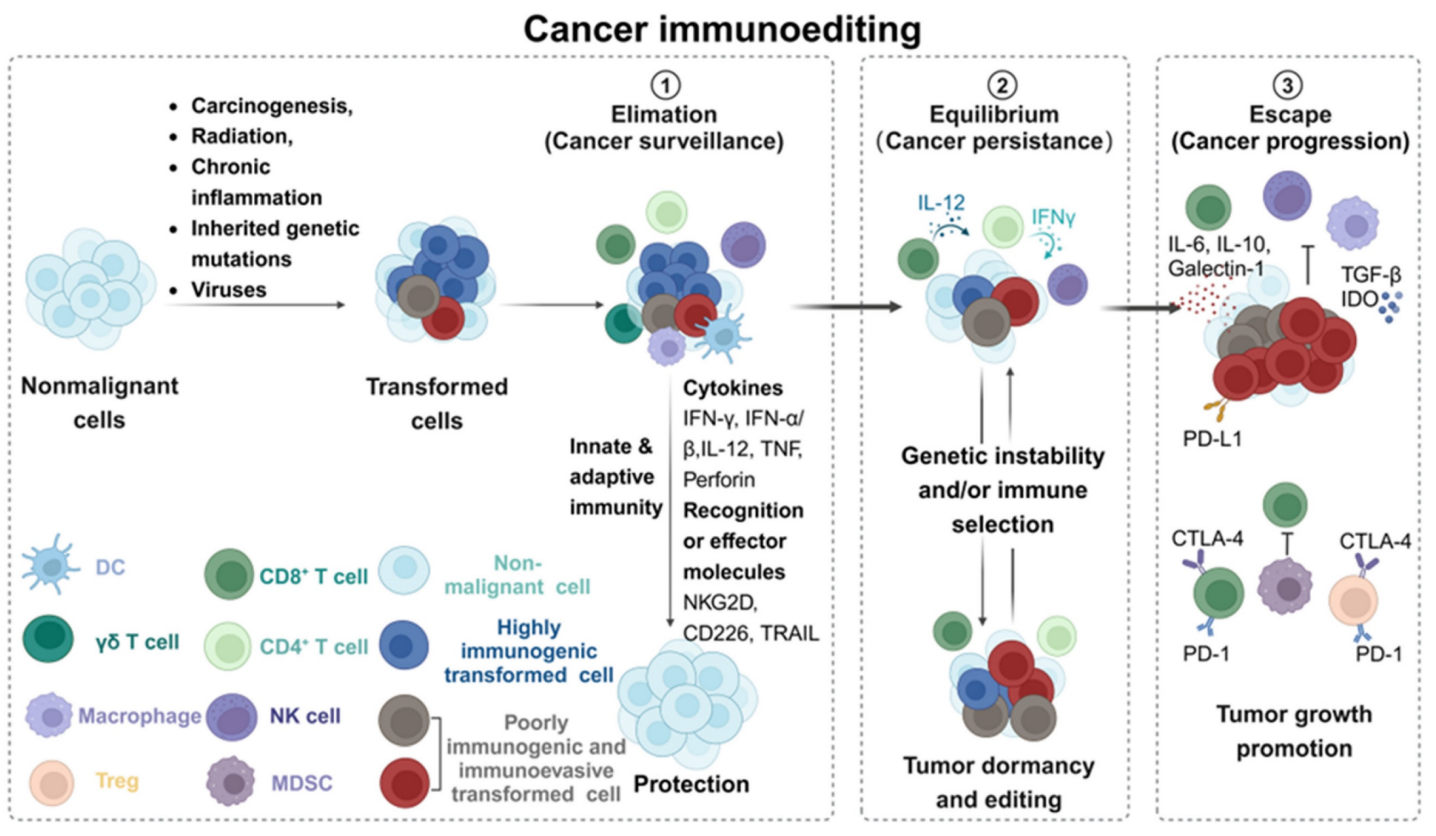
The hypothesis of cancer immunoediting. Treg, regulatory T cell; NK, natural killer; MDSC, myeloid-derived suppressor cell; DC, dendritic cell; IFN-γ, interferon-γ; IFN-α/β, interferon-α/β; IL-12, interleukin-12; TNF, tumor necrosis factor; NKG2D, natural killer cell group 2D; CD226, recombinant cluster of differentiation 226; TRAIL, recombinant tumor necrosis factor related apoptosis inducing ligand; IL-6, interleukin-6; IL-10, interleukin-10; TGF-1β, transforming growth factor-1β; IDO, indoleamine-2,3-dioxygenase; PD-L1, programmed cell death-ligand 1; PD-1, programmed cell death protein 1; CTL-4, cytotoxic T lymphocyte-associated antigen-4. This figure was created with Biorender.com.

The interaction between cancer cells and the immune system is a complex and dynamic process. The cancer immunoediting theory provides a foundational framework for comprehensively understanding this process. Cancer immunoediting 126 is a theoretical construct that elucidates the intricate interplay between cancer cells and the immune system, detailing the evolutionary trajectory of tumors under immune scrutiny and their acquisition of mutations that facilitate evasion of immune detection. This process is characterized by three distinct phases: elimination, equilibrium, and escape **(Figure 9)**. During the elimination phase, the immune system's surveillance function can effectively identify and eliminate precancerous cells or early cancer cells to prevent tumor formation 127. However, some cancer cells can evade immune clearance temporarily and enter the equilibrium stage by employing various mechanisms, such as altering the antigen expression pattern or activating the immunosuppressive pathways. At this stage, the immune system and cancer cells reach a dynamic equilibrium state. The immune system restricts the tumor growth but fails to eradicate it entirely. With the passage of time, cancer cells continue to accumulate genetic and epigenetic variation, and some oof which have acquired a stronger ability to evade immune surveillance. Ultimately, some cancer cells successfully evade the immune system's attack and develop resistance to elimination through immune evasion mechanisms, including the expression of immunosuppressive molecules, secretion of immunosuppressive factors, induction of immunosuppressive cell infiltration, and inhibition of immune cell function through metabolic competition. At this stage, cancer cells can proliferate uncontrollably and eventually develop into tumors with clinical manifestations.

Cancer immunoediting underscores the role of the immune system in inhibiting tumor growth and reveals the tumor's ability to adapt to immune pressure through continuous evolution. This concept is highly significant for understanding the mechanism of cancer occurrence and development, and provides a theoretical basis for the design of novel immunotherapy strategies. Through the intervention of critical steps in the process of cancer immunoediting process, such as enhancing tumor antigen presentation, blocking the immunosuppressive signals, activating immune effector cells, or improving the cancer niches, it is anticipated that the effectiveness of immunotherapy will be heighted, leading to effective tumor control.

Therefore, the interaction between cancer cells and the immune system is a crucial area in tumor biology, offering substantial implications for understanding tumor development, devising effective treatment strategies, and enhancing immunotherapy methods. Further exploration in this field will help to develop new anti-cancer strategies and enhance the efficacy of immunotherapy.

## 5. Other cells metabolism

### 5.1. Cancer-associated fibroblasts (CAFs) metabolism

Cancer-associated fibroblasts (CAFs) play a critical role in tumor growth and invasion, with metabolic reprogramming serving as a key mechanism for their enhanced functionality 128. CAFs exhibit significant versatility and adaptability, contributing to cancer progression through complex interactions with various cell types within the cancer niches 129. In the terms of their metabolic functions, CAFs are responsible for synthesizing extracellular matrix components that enhance the structural integrity and functionality of the tumor matrix. Furthermore, they undergo epigenetic modifications that facilitate the production of secretory factors, exosomes, and metabolites, which subsequently influence tumor angiogenesis, immune responses, and metabolic processes 130.

CAFs significantly enhance the uptake and metabolism of glutamine through metabolic reprogramming, thereby providing essential support for tumor development. During this process, CAFs exhibit a marked dependence on glutamine. Glutamine serves not only as a crucial substrate for protein synthesis but also as a primary pathway for tumor cells to conduct energy metabolism and biosynthesis. Research indicates that CAFs facilitate the uptake and metabolism of glutamine through a series of intricate molecular mechanisms, including the activation of specific transporters and enzymes. This metabolic reprogramming not only meets the energy requirements of CAFs themselves but also influences the metabolic state of adjacent cancer cells by secreting metabolic byproducts and growth factors, thereby promoting the malignant behavior of the tumor. The metabolic reprogramming of CAFs can also generate high levels of energy-rich substrates such as pyruvate, ketone bodies, fatty acids, and lactate through aerobic glycolysis. These products can subsequently stimulate cancer cells and promote their proliferation 131. For instance, acetate released by CAFs in pancreatic cancer has the capacity to alter polyamine metabolism via the ACSS2-SP1-SAT1 signaling pathway, thereby facilitating the advancement of pancreatic cancer 132. Furthermore, CAFs can enhance the survival of cancer cells within an acidic microenvironment by modulating the epigenomic and transcriptomic profiles of these cells.

CAFs can significantly influence the tumor microenvironment by secreting exosomes and cytokines. Exosomes are small vesicles released by cells that contain proteins, nucleic acids, and other bioactive substances 133. The exosomes secreted by CAFs can transport specific metabolites and signaling molecules, promoting tumor proliferation, invasion, and metastasis by directly interacting with cancer cells or modulating the function of immune cells. For instance, the exosomes released by CAFs can enhance tumor metastasis and chemotherapy resistance by augmenting the stem cell characteristics and epithelial-mesenchymal transition (EMT) of colorectal cancer 134. Concurrently, cytokines secreted by CAFs, such as interleukins (ILs) and chemokines, can regulate the immune response and angiogenesis within cancer niches, thereby creating favorable conditions for tumor growth and invasion 135.

The interaction between CAFs and tumor cells significantly influences tumor immune regulation. CAFs can modulate the immune response within the TME by secreting cytokines and chemokines, thereby affecting tumor growth and metastasis 136. In addition, CAFs can regulate the metabolic state of tumor cells by altering the levels of metabolites in cancer niches, such as lactic acid and ATP, which in turn impacts the tumor's invasive and metastatic capabilities. These findings present new targets and therapeutic strategies for future cancer treatments. By disrupting the metabolic reprogramming and secretory functions of CAFs, it may be possible to inhibit tumor growth and invasion, thereby enhancing the efficacy of cancer therapies.

### 5.2. Endothelial cells metabolism

Endothelial cells are the primary cell type found in the inner lining of blood vessels, which are crucial for maintaining the integrity and functionality of these vessels 137. They meet the material and energy demands necessary for angiogenesis through metabolic remodeling. Glucose metabolism, amino acid metabolism, fatty acid metabolism, and other metabolic pathways each play distinct roles in angiogenesis 138. In cancer niches, the metabolic reprogramming of vascular endothelial cells (VECs) is vital for tumor angiogenesis. Through this metabolic reprogramming, VECs satisfy the material and energy requirements essential for tumor angiogenesis 139. Various metabolic pathways, including glucose metabolism, amino acid metabolism, and fatty acid metabolism, contribute differently to the process of tumor angiogenesis.

Metabolic reprogramming of VECs involves changes at multiple levels. First, the metabolic status of VECs within cancer niches is as crucial as the stimulation of angiogenic factors. For instance, glucose metabolism in VECs is predominantly relies on the glycolytic pathway, which serves as the primary energy source, while fatty acid metabolism primarily contributes to biosynthesis 140. Furthermore, both glutamine and asparagine metabolism are significant energy sources for VECs.

In the process of tumor angiogenesis, the metabolic remodeling of VECs is significant. Compared to normal endothelial cells, VECs in tumor tissues undergo various metabolic changes, including enhanced glycolysis, increased fatty acid oxidation, and upregulation of glutamine metabolism. For instance, fructose metabolism promotes angiogenesis in tumor endothelial cells by activating AMPK signaling and enhancing mitochondrial respiration 141. Furthermore, the metabolic reprogramming of VECs is also associated with immune regulation and extracellular matrix formation within the tumor microenvironment. The metabolic status of VECs can influence their response to signals emitted by tumor cells, thereby facilitating new blood vessel formation. This metabolic reprogramming is crucial for supplying nutrients and enhancing drug delivery to tumors, as angiogenesis provides essential nutrients and oxygen for tumor growth and creates pathways for tumor metastasis 142.

## 6. Emerging therapeutic strategies in cancer metabolism

### 6.1. Dietary intervention

**Figure 10:**
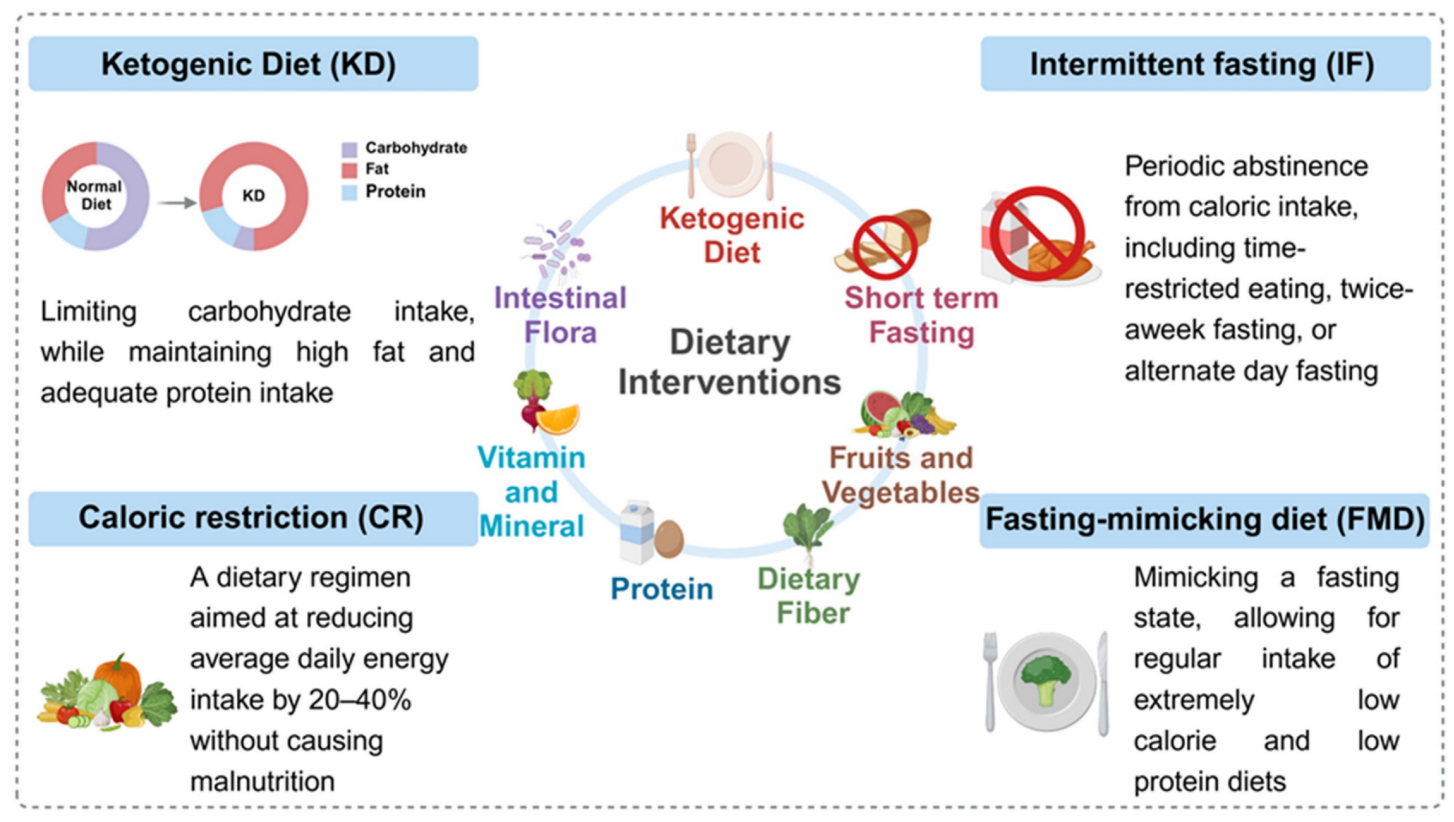
Classification of dietary interventions. Dietary interventions, such as caloric restriction (CR), Intermittent fasting (IF), and ketogenic diet (KD), can modulate the progression and treatment sensitivity of various diseases, including cancer. This figure was created with Biorender.com.

Understanding the metabolic pathways of glucose, lipids, and amino acids has established a fundamental basis for investigating the effects of dietary interventions on tumor therapy 143, 144. Macronutrients, including carbohydrates, fats, and proteins, are the primary sources of energy in the human body. Each nutrient follows a unique metabolic pathway to provide essential energy and nutrients for the body. Modulating the consumption of these nutrients can impact their utilization within the body, consequently influencing overall metabolism. This concept is at the core of implementing various dietary restrictions and special dietary strategies. Whether through the adoption of a ketogenic diet, an amino acid-specific diet, caloric restriction, or intermittent fasting **(Figure 10)**, these dietary approaches can alter the body's metabolic state by adjusting nutrient intake to achieve specific health goals. The utilization of these dietary strategies hinges on a comprehensive comprehension of the metabolic pathways of glucose, lipids, and amino acids.

A common dietary intervention strategy is a low-carbohydrate diet, such as the ketogenic diet 145. This dietary approach reduces the energy source available to cancer cells through the restriction of carbohydrate consumption and the reduction of blood glucose levels. Because cancer cells often heavily rely on glycolysis for energy, restricting carbohydrate intake can selectively deprive cancer cells of energy and minimize the impact on normal cells. The ketogenic diet can also modify the cancer niches, diminish the inflammatory response, and potentially inhibit tumor growth by increasing the production of ketones.

Another strategy is to limit the intake of specific amino acids, such as methionine. Methionine is an essential amino acid for the growth of cancer cells 146. By limiting methionine intake, the growth of cancer cells can be selectively impeded with minimal impact on normal cells. This dietary intervention has shown an inhibitory effect on tumor growth in some preclinical studies. Furthermore, caloric restriction and intermittent fasting are established dietary strategies that have been extensively investigated. Caloric restriction can reduce the metabolic rate of cancer cells by decreasing energy intake, thereby decelerating tumor growth. Intermittent fasting 147 may inhibit tumor growth by triggering autophagy and diminishing the levels of growth factors such as insulin-like growth factor-1 (IGF-1).

In addition to directly affecting cancer metabolism, dietary intervention can also influence tumor growth and metastasis by regulating the immune system. For instance, certain dietary ingredients exhibit anti-inflammatory properties that can diminish the inflammatory reaction in the cancer niches, thereby reducing tumor growth and metastasis. In addition, some nutrients, such as vitamins and minerals, can also enhance the function of the immune system and improve the immune response to tumors.

As a new approach to disease management, dietary intervention shows promise for both research exploration and clinical application value. However, the effectiveness and safety of these strategies still need to be rigorously investigated. With further research, dietary intervention may become an integral part of personalized medicine, offering a novel approach to prevent and treat numerous diseases.

### 6.2. Targeted cancer metabolic therapy

Targeted cancer metabolism is an important therapeutic strategy in the era of precision oncology. It is based on the metabolic differences between cancer cells and normal cells and aims to control cancer development by interfering with the specific metabolic pathways of cancer cells. Cancer cells often adopt different metabolic pathways from normal cells to meet their rapid proliferation's energy and material demands. For instance, cancer cells commonly rely on glycolysis to metabolize glucose and generate substantial amounts of lactic acid even in oxygen-rich environments. By focusing on this distinct metabolic phenotype of cancer cells, therapeutic interventions can be developed to modulate their metabolism and effectively manage cancer. In recent years, an increasing number of research investigations have elucidated the intricate interconnection between the metabolic processes of cancer cells and the immune system response. Targeting cancer metabolism can not only directly inhibit the energy supply and material synthesis of cancer cells, thereby slowing down their growth rate, but also affect the activity and function of immune cells by regulating the cancer niches.

#### 6.2.1. Targeted cancer cell metabolism

Targeting cancer cells primarily involves the specific metabolic pathways and enzymes of cancer cells 75, 148. Given the rapid proliferation rate and active nucleotide synthesis characteristic of cancer cells, the disruption of nucleotide synthesis has emerged as a critical strategy for anti-tumor therapy 58, 149. Various approaches can be employed to interfere with nucleotide synthesis, including the inhibition of folate metabolism, competitive inhibition using nucleotide analogs, and blocking essential synthases 150. Folic acid is an essential cofactor in nucleotide synthesis, which can be effectively disrupted by interfering with the metabolism of folic acid. Inhibitors of dihydrofolate reductase, such as methotrexate, inhibit the synthesis of DNA and RNA by irreversibly binding with dihydrofolate reductase to prevent the production of tetrahydrofolate 151. At present, the drug is widely used in cancer treatment, such as acute leukemia and malignant lymphoma, as well as autoimmune diseases like rheumatoid arthritis. In addition, some drugs can inhibit nucleotide synthase, thereby blocking the DNA replication and RNA transcription of cancer cells, ultimately inhibiting the growth of tumors. Nucleotide synthase is the key enzyme responsible for synthesizing nucleotides in cells, which is essential for maintaining the normal cellular physiological function., Gemcitabine, an important anti-cancer drug, is widely used in the treatment of pancreatic cancer, non-small cell lung cancer, breast cancer, ovarian cancer, and other solid tumors 152. Gemcitabine can be converted into active nucleoside diphosphate (dFdCDP) and nucleoside triphosphate (dFdCTP) by intracellular nucleases. Among them, dFdCDP can inhibit the activity of nucleotide reductase, reducing the supply of deoxyriboside triphosphate, which is the raw material for DNA synthesis in cells. This inhibition ultimately hinders the normal synthesis and repair of DNA, leading to cell apoptosis. In addition, fluorouracil is converted into the active metabolite fluorodeoxyuridine triphosphate (FdUMP) in the body. It forms a stable ternary complex with thymidine nucleotide synthase (TS) and reduced folic acid, thereby blocking the conversion of uracil nucleotides to thymine nucleotides and affecting DNA synthesis 153.

Cancer cells often exhibit the characteristics of metabolic reprogramming to sustain rapid growth and survival. This includes acceleration the glycolytic process (known as the Warburg effect), increased reliance on specific amino acids, and modifications in lipid metabolism. These changes in metabolic pathways provide potential therapeutic targets for targeted therapy **(Table 2)**. For example, by inhibiting the glycolysis process, such as through the use of 2-deoxyglucose (2-DG) to impede glucose transport and phosphorylation, the energy supply to cancer cells can be constrained, thereby hindering their proliferation and survival 154. Furthermore, cancer cells often exhibit an excessive dependence on certain amino acids, such as glutamine. To address this dependency, various inhibitors have been developed. For instance, CB-839 is a small molecule that inhibits glutaminase, thereby blocking cancer cell's ability to utilize glutamine for biosynthesis and energy production 155. In addition, targeted lipid metabolism is also a promising field. Cancer cells often adjust their lipid synthesis and decomposition pathways to support membrane synthesis and energy requirements. Inhibition of key lipid metabolic enzymes, such as fatty acid synthase, can weaken the viability of cancer cells.

#### 6.2.2. Targeted cancer niches metabolism

The ongoing advancement in the exploration of the cancer niches has enabled the potential targeting of the specific components of the cancer niches. The cancer niche is composed of cancer cells and their surrounding immune cells, vascular cells, extracellular matrix, and other cell types. Due to the hypoxic state, lack of nutrients, and highly acidic environment of cancer niches, the efficacy of immune cells such as T cells and natural killer cells is often suppressed, limiting the effectiveness of immunotherapy.

The high glycolytic activity of cancer cells leads to a decrease in glucose levels in cancer niches, impacting the activity and function of immune cells. The normal immune function can be restored by targeting the cancer niches in a low-glucose environment. The glycolysis of cancer cells can be reduced by inhibiting glycolytic regulatory enzymes or using competitive glucose analogue 2-DG. This approach can effectively decrease the proliferation of cancer cells and promote the formation of long-term memory CD8^+^ T cells.

Lactic acid is an important metabolite in cancer niches, playing a crucial role in tumor immune evasion and immunosuppression. The increase in lactic acid levels can alter the activity and differentiation of immune cells. Therefore, targeting lactate response pathways or receptors, such as GPR81, may provide a promising approach for cancer treatment. By inhibiting the function of lactate transporters such as MCT1 and MCT4, the accumulation of lactate in cancer niches can be reduced, thereby decreasing its inhibitory effect on immune cells 187. In addition, the production and transformation of lactic acid in cancer niches can be controlled by regulating the activity of lactate dehydrogenase (LDH). Additionally, alleviating immunosuppression by inhibiting immune checkpoint molecules like PD-1/PD-L1 and CTLA-4 can enhance the cytotoxicity of immune cells against cancer cells 188. These inhibitors are extensively utilized in the clinical management of diverse tumors.

Immune metabolism offers a new opportunity to identify new therapeutic targets and enhance cancer treatment by regulating cancer niches. Although an in-depth study of the single metabolic pathway in cancer or immune cells can enhance the understanding of the metabolism mechanism in cancer niches, it is challenging to fully grasp the complexity of the cancer niches by solely concentrating on a single enzyme or transporter within a specific pathway. Therefore, the combination of metabolic targets and traditional chemotherapy or targeted intervention therapy (such as immune checkpoint blockade) is the key to improving clinical efficacy.

## 7. Cell metabolism in non-neoplastic diseases

Cell metabolism, a fundamental process of life, is essential not only for energy supply and biosynthesis but also plays a critical role in the initiation and progression of complex diseases. In the field of cancer, metabolic reprogramming has emerged as one of the hallmark features. Cancer cells satisfy their energy and biosynthetic demands for rapid proliferation by enhancing glycolysis, glutamine catabolism, and other metabolic pathways. This metabolic transformation not only promotes tumor growth but also influences the sensitivity and resistance of tumors to treatment. Furthermore, cell metabolism is also significant in the initiation and progression of non-neoplastic diseases, including cardiovascular diseases, neurodegenerative disorders, and metabolic syndrome. In these instances, abnormal activation or inhibition of metabolic pathways can lead to pathological changes.

In cardiovascular diseases, abnormal activation of metabolic pathways can significantly contribute to the development of atherosclerosis and cardiomyopathy 189. For instance, the disruption of lipid metabolism may result in the accumulation of lipids within the vascular wall, leading to plaque formation and subsequently causing atherosclerosis 190. Cardiac metabolic remodeling, which encompasses the metabolic reprogramming of cardiomyocytes, is essential for maintaining cardiomyocyte homeostasis 191. Adverse remodeling is typically triggered by the combined effects of various stressors, such as obesity, diabetes, and hypertension. Therefore, the regulation of metabolism and the underlying causes of metabolic disorders are focal points in life sciences and medical research.

Furthermore, in the context of neurodegenerative diseases such as Alzheimer's disease and Parkinson's disease, mitochondrial dysfunction and alterations in metabolic pathways are significantly associated with diseases progression 192. For instance, in Alzheimer's disease, impaired glucose metabolism within the brain during can lead to neuronal dysfunction and a sustained energy deficit, ultimately resulting in neuronal impairment and the accumulation of neurotoxic proteins. This cascade of events contributes to cognitive decline and the emergence of neuropsychiatric symptoms 193. Furthermore, lactic acid modification is prevalent in the brain in the context of neurodegenerative diseases, playing a crucial role in the regulation of in chromatin status and gene expression. Such modifications may be influenced by neuronal excitation and social stress, which are linked to reductions in social behavior and increases in anxiety-like behavior 194. In addition, lactate plays multiple roles in the recovery process following myocardial infarction, which may promote cardiac fibrosis by activating the TGF-β signaling pathway or facilitate cardiac repair by promoting the early activation of monocytes 195.

In metabolic diseases such as type 2 diabetes mellitus, cellular metabolic reprogramming significantly influences insulin sensitivity and glucose metabolism 196. Unlike the absolute insulin deficiency observed in type 1 diabetes, type 2 diabetes mellitus is characterized by insulin resistance and hyperglycemia, resulting from relative insulin deficiency. Patients with type 2 diabetes mellitus frequently exhibit metabolic abnormalities, report fatigue, and faced an increased risk of cardiovascular and cerebrovascular diseases. In addition, in metabolic disorders, lactation is associated with obesity and insulin resistance, which may impact the metabolic pathways of skeletal muscle and white adipose tissue. In the context of inflammatory and infectious diseases, lactation is closely linked to macrophage differentiation, potentially influencing the expression of tissue repair genes.

Therefore, the potential effects of cellular metabolism in various diseases are multifaceted, encompassing energy production, biosynthesis, signal transduction, and cell death. Understanding the molecular mechanisms of these metabolic changes is crucial for developing new therapeutic strategies. For example, drugs that target mitochondrial metabolism may offer significant therapeutic benefits for cancer and neurodegenerative diseases 189. Furthermore, enhancing the function of immune cells by regulating metabolic pathways may be beneficial for autoimmune disorders and certain inflammatory diseases.

## 8. Clinical trials targeting cancer metabolism

Currently, clinical trials around the globe are actively investigating novel therapies that target the distinct metabolic characteristics of cancer cells **(Table 3)**. These studies aim to enhance the effectiveness of cancer treatment while minimizing side effects of drugs for patients.

The clinical trial results for the KRAS G12C-targeted drug D-1553 are remarkable. This drug is specifically designed for patients with non-small cell lung cancer (NSCLC) harboring the KRAS G12C mutation and functions by inhibiting the activity of the KRAS G12C protein, thereby impacting the growth and metabolism of cancer cells 204. In Phase II clinical trials, D-1553 demonstrated a high tumor remission rate, prolonged remission duration, and a favorable safety profile 205. Specifically, the objective response rate (ORR) was 50%, the disease control rate (DCR) was 89%, the median duration of response (DOR) was 12.8 months, and the median progression-free survival (PFS) was 7.6 months. These findings suggest that D-1553 has the potential to be an effective treatment for KRAS G12C mutant NSCLC.

Enhertu (trastuzumab deruxtecan) is an antibody-drug conjugate (ADC) developed by Daiichi Sankyo using its proprietary DXd ADC technology 206. By combining a humanized HER2 monoclonal antibody with a topoisomerase I inhibitor payload, it facilitates precise delivery to cancer cells. In the Phase Ⅲ clinical study (DESTINY-Breast06) involving metastatic breast cancer patients with low HER2 expression and positive hormone receptor (HR) status, Enhertu demonstrated favorable outcomes. Specifically, in the DESTINY-Breast06 study, Enhertu significantly improved PFS compared to standard chemotherapy. Notably, Enhertu reduced the risk of disease progression or death by 38%, with a median PFS of 13.2 months, compared to 8.1 months in the chemotherapy group. The ORR for patients with low HER2 expression was 56.5%, while the ORR in the chemotherapy group was 32.2%. The safety profile of Enhertu aligns with findings from previous clinical trials in breast cancer, and no new safety concerns have been identified. These data suggest that Enhertu has the potential to serve as a new treatment option for patients with HER2 low-expression metastatic breast cancer, particularly those who have undergone one or more endocrine therapies. The research findings were presented at the American Society of Clinical Oncology (ASCO) annual meeting in 2024 and have been submitted to global regulatory authorities.

Furthermore, Trodelvy (sacituzumab govitecan), an ADC targeting Trop-2, is currently undergoing a Phase Ⅲ clinical trial for second-line metastatic bladder cancer (TROPiCS-04). Trop-2 is a cell surface antigen that is highly expressed in various tumor types and is closely associated with the proliferation, migration, and metabolism of cancer cells 207. Trodelvy targets Trop-2, delivering the topoisomerase inhibitor SN-38 to cancer cells that express Trop-2, thereby interfering with their metabolic pathways and playing a therapeutic role. This trial was designed to compare the overall survival of patients with metastatic or locally advanced unresectable urothelial carcinoma treated with Trodelvy against a treatment regimen selected by their physicians. According to the latest news released by Gilead Sciences on May 30, 2024, the TROPiCS-04 study did not achieve its primary endpoint. Specifically, there was no significant difference in overall survival (OS) within the intention-to-treat (ITT) population. Nevertheless, the Trodelvy treatment group demonstrated a numerical improvement in OS and exhibited a positive trend in certain predefined subgroups and secondary endpoints, such as PFS and ORR. Additionally, the number of treatment-related deaths was higher in the Trodelvy group, primarily due to complications associated with neutropenia, including infections. Although Trodelvy did not meet the primary endpoint in the TROPiCS-04 study, the data suggest that it may offer clinical benefits for specific patient subgroups.

In the treatment of endometrial and ovarian cancer, the combination therapy of Jemperli and Zejula has shown promising prospects. These two drugs target the immune evasion and DNA repair mechanisms of cancer cells by blocking the PD-1 receptor and inhibiting PARP enzyme activity, thereby disrupting their metabolism and growth. Jemperli (dostarlimab) is a PD-1 inhibitor antibody that binds to the PD-1 receptor, blocking its interaction with the ligands PD-L1 and PD-L2 208, 209. This action alleviates the suppression of the immune system and enhances the ability of T cells to attack tumor cells. Zejula (niraparib) is an oral PARP inhibitor that promotes cancer cell death by inhibiting the PARP-mediated DNA damage repair process. The combination therapy of these two drugs is currently undergoing clinical research for various types of cancer. In the second part of the RUBY Phase Ⅲ clinical study, the combination of Jemperli and Zejula improved the primary endpoint of PFS in patients with primary advanced or recurrent endometrial cancer. These results support the potential of Jemperli and Zejula combination therapy in the treatment of endometrial cancer. Regarding the treatment of ovarian cancer, the combination therapy of Jemperli and Zejula was initially evaluated in a Phase Ⅲ study. Although the specific research results have not yet been published, existing information indicates that this study aims to assess the efficacy and safety of this combination therapy in patients with ovarian cancer. While the clinical trials show promising signs, the detailed research outcomes, including OS and safety data, are still pending the publication of the final clinical research results. Furthermore, the approval and use of any drug must adhere to the guidelines and approvals of the relevant regulatory authorities.

Another noteworthy clinical trial is the combination therapy of tiragolumab and Tecentriq. These two drugs target the TIGIT and PD-L1 immune checkpoints, respectively, and influence the immune microenvironment and metabolic state of cancer cells by regulating the activity and function of immune cells 210. Tiragolumab is a monoclonal antibody that specifically targets TIGIT. It blocks the interaction between TIGIT and CD155 by binding to the TIGIT receptor, which may enhance the body's immune response. Tecentriq (atezolizumab) is a monoclonal antibody that targets PD-L1, restoring the anti-tumor activity of T cells by inhibiting the binding of PD-L1 to PD-1. In the Phase Ⅲ clinical study SKYSCRAPER-01, the combination therapy of tiragolumab and Tecentriq was evaluated as a first-line treatment for patients with locally advanced or metastatic NSCLC exhibiting high PD-L1 expression. Preliminary analysis indicated that, compared to Tecentriq monotherapy, the combination therapy demonstrated a trend toward improved OS. However, the data were not yet mature and did not achieve statistical significance. Nevertheless, these findings offered a novel perspective on the use of combination therapy with immune checkpoint inhibitors and may inform future treatment strategies. Although a numerical improvement was observed in the preliminary analysis, the OS data remain immature, necessitating further data analysis and publication for conclusive results. Additionally, the safety and tolerability of the tiragolumab and Tecentriq combination therapy are critical components of the study. Preliminary data suggest that the combination therapy is well tolerated, with no new safety signals identified upon the addition of tiragolumab. Overall, the preliminary results of the combination therapy involving tiragolumab and Tecentriq in the SKYSCRAPER-01 study are promising. The final analysis of OS results is still pending to ascertain whether this combination therapy can statistically and significantly enhance the survival of patients with locally advanced or metastatic NSCLC exhibiting high PD-L1 expression.

Finally, as a targeted therapy for the NTRK fusion gene, entrectinib has demonstrated significant efficacy in several clinical trials 211. The NTRK fusion gene can cause cancer cells to produce abnormal TRK proteins, thereby activating downstream signaling pathways that promote the growth and metabolism of cancer cells. In a clinical study involving patients with NTRK fusion-positive advanced solid tumors, the PFS and median OS for entrectinib were 13.8 months and 33.8 months, respectively. Additionally, entrectinib has shown a significant impact on intracranial metastasis, offering a new treatment option for patients with NTRK fusion positivity. Furthermore, entrectinib exhibited favorable outcomes in patients with tumors that metastasized to the brain, achieving an objective intracranial remission rate of 54.5%, with a quarter of these patients attaining complete remission. In another study, the PFS and median OS of NTRK fusion-positive solid tumors treated with entrectinib were 13.8 months and 33.8 months, respectively. This indicates that entrectinib is not only effective for patients with solid tumors but can also significantly improve the survival time of patients with intracranial metastasis. These efficacy data for entrectinib provide strong evidence for its application in the treatment of NTRK fusion-positive solid tumors and offer new treatment options for patients.

In summary, the current clinical trials related to targeted cancer metabolism encompass a diverse array of drugs and targets, including KRAS G12C, HER2, Trop-2, PD-1/PD-L1, TIGIT, and NTRK fusion genes. These studies not only offer new treatment options for cancer patients but also provide valuable data that supports a deeper understanding of cancer metabolic mechanisms.

## 9. Challenges and Opportunities

However, there are still multiple challenges in using the metabolism of cancer cells and immune cells to guide cancer therapy. The immaturity of diagnostic technologies is a key issue. Although some detection technologies can be used to detect metabolic changes in tumors, there is still a need to improve their sensitivity, specificity, and accuracy 212. Moreover, although immunohistochemistry and flow cytometry are used to assess the status of immune cells, these strategies typically require professional researchers to operate and analyze, and they are time-consuming, limiting their widespread application. Moreover, the existing imaging technology cannot accurately monitor the metabolic interactions between cancer cells and immune cells in real-time, often missing the window of opportunity to utilizing cancer metabolism process to guide treatment. At the same time, the lack of cancer-specific and sensitive metabolic markers poses significant challenges for early diagnosis of cancer and timely evaluation of treatment effectiveness. Therefore, there is an urgent need to develop more convenient, precise, and reliable diagnostic technologies in the field of cancer metabolism. Furthermore, multidrug resistance (MDR) is another major challenge in cancer treatment. When cancer cells develop resistance to one chemotherapy drug, they often exhibit cross-resistance to other chemotherapy drugs with different chemical structures and mechanisms of action, making cancer treatment extremely challenging. The mechanisms of MDR in cancer cells are complicated and diverse, involving the increased drug efflux pumps, alterations in drug targets, and the inhibition of apoptotic pathways 213. Therefore, overcoming the issue of MDR in cancer cells requires a multi-faceted approach in utilizing cancer metabolism-based therapeutic strategies. This includes developing new drug targets, identifying therapeutic agents capable of reversing multidrug resistance, and exploring novel combination treatment strategies.

The heterogeneity of cancer and individual differences among patients also present challenges in cancer treatment. Typically, there are variations in cancer phenotype, immune system status, and patient physiology among individuals, resulting in vastly different treatment outcomes for the same therapeutic approach in different patients. An alternative strategy is to develop personalized cancer treatment approaches. To achieve personalized treatment, we need to consider a comprehensive range of factors including patients' clinical data, cancer progression status, tumor genomic information, immune cell status, and immunohistochemistry data for formulating the most suitable treatment plan tailored to individual patient. Moreover, the regulation of cancer metabolism and immune system response for cancer treatment requires comprehensive consideration of multiple factors, such as the supply of nutrients and energy, regulation of metabolic pathways, activation of immune cells, and secretion of cytokines and chemokines. These factors interact with and constrain each other, resulting in highly complicated cancer treatment plans along with suboptimal therapeutic efficacy. Furthermore, the occurrence, growth, recurrence, and metastasis of cancer is a dynamic process, requiring continuous adjustments to the treatment formulation during therapy to suppress cancer progression.

Given that there are still multiple challenges in using the metabolism of cancer cells and immune cells to guide clinical cancer treatment, future research directions in cancer metabolism therapy may focus on innovations in diagnostic technologies, deepening understanding of metabolic mechanisms, expansion of clinical sample analysis databases, and personalized treatment. For example, more comprehensive cancer metabolism data can be obtained through carefully designed clinical trial protocols and the conduct of additional clinical trials, benefiting a broader patient population. These clinical trials will encompass studies at various stages of cancer, ranging from initial safety assessments to large-scale efficacy validations. By collecting and analyzing this clinical data, we can more accurately evaluate the safety and effectiveness of treatment strategies, thereby providing strong evidence for the clinical application of cancer metabolism therapies. Moreover, an in-depth exploration of the interactions between cancer cells and the immune system will aid in developing more effective cancer metabolism treatment strategies. This method includes understanding how cancer cells evade immune surveillance, how immune cells are activated, and how the immune system recognizes and kills cancer cells. By exploring the immune mechanisms within cancer niches, we can identify new drug targets and therapeutic strategies to enhance the antitumor activity mediated by immune cells.

Considering the heterogeneity of cancer, future cancer treatment strategies will lean towards personalized therapy. By combining the individual patient's genomics, metabolomics, immune status, and other relevant information, we can develop a unique treatment formulation for individual patient. This personalized medical strategy will help improve the effectiveness of cancer therapy and reduce side effects. Moreover, advanced diagnostic equipment is essential for real-time monitoring and evaluating the effectiveness of treatments. With the advancements in cancer imaging technology and biomarkers, it will become possible to monitor cancer growth, metastasis, and timely assess the effectiveness of treatment strategies in real-time. Additionally, these technologies can also be used to evaluate the patient's immune status and metabolic changes, providing crucial references for developing safe and reliable cancer treatment plans. Furthermore, using the metabolism of cancer cells and immune cells to guide cancer treatment requires interdisciplinary collaboration and cooperation. By fostering cooperation and academic exchanges between various disciplines, we can enhance the sharing of fundamental research and clinical application data, stimulate new ideas for innovative cancer treatments, and collectively advance the rapid development in the field of cancer metabolism.

## 7. Conclusion

In exploring new frontiers in cancer treatment, understanding the interaction between cancer cells and immune cells within metabolic pathways in the cancer niches helps identify potential therapeutic targets and elucidate disease progression. Studies have shown that the metabolic adaptability of cancer cells not only supports their rapid proliferation and survival but also indirectly inhibits the activity and function of immune cells by altering the metabolic state of cancer niche.

Firstly, the metabolic reprogramming mechanisms of cancer cells suggest that targeting specific metabolic processes of cancer cells, such as glycolysis and the activation of certain metabolic enzymes, can directly weaken the proliferative activity of cancer cells. Secondly, regulating the metabolic state of anti-tumor immune cells, such as cytotoxic T lymphocytes, by promoting their glycolysis and fatty acid oxidation processes, can significantly enhance T cell-mediated cancer cell killing. Thirdly, modulating the metabolic characteristics of the cancer niches, such as altering its pH, reducing hypoxia, or restricting the supply of nutrients (such as amino acids), can also provide new methods for cancer metabolism mediating-therapy. Moreover, combining immune checkpoint blockade with cancer metabolism regulation will offer new approaches to improve response rates of cancer immunotherapy and prolong patient's survival period.

In summary, the study on the metabolic pathways of cancer cells and immune cells not only deepens the understanding of the mechanisms of cancer occurrence, development, and metastasis but also provides reliable theoretical guidance for developing new cancer treatment strategies. Given the heterogeneity of cancer, future scientific research needs to further explore the roles of cancer metabolic pathways in different types of cancer and individual patients, as well as how to leverage these metabolic pathways to identify new targets for guiding safe and effective clinical cancer treatments, which will bring more hope and change to patients with advanced cancer.

## Figures and Tables

**Table 1:** Main immune cells and their main metabolic pathways in the cancer immune niches

Cell type	Function	Metabolic pathway
*Immune activation or inflammatory*		
NK cell	Non-specific killing of cancer cells and virus infected cells; Release cytotoxins, granzyme and perforin; Secreting anti-tumor cytokines and interferon.	OXPHOS and glycolysis
Inflammatory TAM	Release of proinflammatory cytokines (TNF-α, IL-1β, IL-6); Secretion of chemokines and matrix metalloproteinases	Glycolysis and PPP
DC	Antigen presentation; Activate T cells; Regulating immune response.	OXPHOS and glycolysis
Teff cell	Identify and attack cells or cancer cells infected by pathogens (viruses and bacteria); Release interleukin and interferon.	OXPHOS and glycolysis
Tm cell	Long term antigen memory	OXPHOS and glycolysis
*Immunosuppression*		
MDSC	Inhibit immune cell response ability	OXPHOS and glycolysis; Amino acid metabolism
Immunosuppressive TAM	Release immunosuppressive cytokines (IL-10, TGF-β and arginase-1); Secrete growth factors and chemokines (VEGF, FGF, PDGF); Production of proteases (such as matrix metalloproteinases)	Glycolysis and FAO; Amino acid metabolism
Treg cell	Recognize and inhibit overactivated T cells	OXPHOS

NK, natural killer; OXPHOS, oxidative phosphorylation; TAM, tumor associated macrophage; TNF-α, tumor necrosis factor-α; IL-1β, interleukin-1β; IL-6, interleukin-6; DC, dendritic cell; PPP, pentose phosphate pathway; IL-10, interleukin-10; TGF-β, transforming growth factor-β; VEGF, vascular endothelial growth factor; FGF, fibroblast growth factor; PDGF, platelet-derived growth factor; Teff, effector T cell; Tm, memory T cell; Treg, regulatory T cell; MDSC, myeloid-derived suppressor cell; FAO, FA oxidation.

**Table 2:** Drugs targeting cancer cell metabolism and their research progress

Target	Pathways	Agents	Development stage	Observations	Refs
HK	Glycolysis	2-DG 3-BP Lonidamine Tuvatexib (VDA-1102) C-02 VDA-1275	The clinical development of 2-DG and 3-BP has been discontinued; Lonidamine has been approved for the treatment of cancer. Tuvatexib is in clinical development; The development of C-02 and VDA-1275 is preclinical.	HK, especially HK2, is overexpressed in cancer cells, and its inhibition can block glycolysis; Vidac Pharma reported the positive interim phase 2A data of VDA-1102 in the treatment of orphan disease cutaneous T-cell lymphoma (CTCL) on January 29, 2024.	156
PFKFB3	Glycolysis	3-PO AZ-67 PFK-158	Preclinical data	PFKFB3 is the rate limiting enzyme of glycolysis, and inhibiting it can reduce glycolysis	157
PKM2	Glycolysis	TP-1454 11C-DASA-23 DNX-3000 ML082 ML170 ML265 ML285 PKM2 inhibitors (NIPER, 7d) CAP-232 (TLN-232)	TP-1454 is in clinical development; The clinical development of CAP-232 has been discontinued.	NIPER can be used as a drug to treat triple negative breast cancer by inhibiting PKM2. PKM2 indirectly affects the TCA cycle by regulating the generation of pyruvate	158-161
LDHA	Glycolysis	Galloflavin GNE-140 RS6212 MS-6105	The development of galloflavin, GNE-140 and RS6212 are preclinical; MS-6105 is in drug discovery stage.	The drug type of MS-6105 is proteolysis targeted chimera	162
GLUT1	Glycolysis	ABSK-006 DRB-18 GLUT1 modulators (compound 8) ICO-33 SMI277 WZB117	Preclinical data	Professor Yan Ning's team first analyzed the crystal structure of human glucose transporter GLUT1 in 2014, initially revealed its working mechanism and the pathogenesis and played an important role in overcoming cancer.	163, 164
GLS/GLS1	Glutamine metabolism	IACS-6274 CB-839 JHU-083 UPGL-00004 JAB-24114 CPU-L1 HYL-001 IPN60090	IACS-6274 and CB-839 are in clinical development; The clinical development of IPN60090 has been discontinued.	Inhibition of glutaminase-1 (GLS-1) hampers the proliferation of tumor cells reliant on glutamine	165, 166
ASCT2	Glutamine metabolism	Idactamab	Idactamab is in clinical development	Inhibition of ASCT2 can significantly inhibit the growth and proliferation of tumor cells, increase oxidative stress injury and cell death, and then promote the anti-tumor response.	167, 168
CPS1	Glutamine metabolism	H3B-120	Preclinical data	The overexpression of CPS1 facilitates pyrimidine synthesis to promote tumor progression in certain cancer types, while in other cancer types, CPS1 function prevents the buildup of toxic levels of intratumoral ammonia to allow for sustained tumor growth.	169
FASN	Lipid metabolism	Denifanstat AB-1015 ASC60 GSK837179A MFI03 TVB-3664 TVB-3166 5-Hydroxy Lansoprazole Sulfide AZ-12756122	Denifanstat and AB-1015 are in clinical development	The inhibition of FASN can affect the signaling pathways related to cancer promoting lipid molecules, thus inhibiting the development of tumor	170, 171
MAGL	Lipid metabolism	11C-MAGL-2-11 11C-PAD 18F-MAGL-4-11 [11C]-RO7284390 [18F]FEPAD	Preclinical data	MAGL affects the malignant behavior of tumor by regulating the level of FFA and other ways. The specific mechanism of MAGL in the occurrence and development of tumor needs further research and exploration.	172
AKT	Lipid metabolism	Capivasertib Afuresertib Dordaviprone GCD-0068 Paxalisib GLR-131 HC0201 HC0301 LY-2780301 MK-2206 OB-318 SM-020 TAS-117 Uprosertib	Capivasertib has been approved by the United States, South Korea, Japan, Australia and Canada in the treatment of HR positive/HER2 negative breast cancer.	Abnormal activation of Akt can promote the survival and growth of cancer cells	173
CPT1	FAO	Perhexiline	Perhexiline in pancreatic ductal adenocarcinoma is in preclinical stage in the United States	Inhibition of CRT1 can inhibit the proliferation and invasion of tumor cells, and may improve the sensitivity of tumor cells to some drugs	174-176
DHFR	Nucleotide metabolism	Methotrexate	Methotrexate has been approved by FDA for the treatment of cancer.	Some anticancer drugs inhibit the proliferation of cancer cells by inhibiting the activity of DHFR interfering with folate metabolism in cells.	177
RNR	Nucleotide metabolism	Gemcitabine Triapine BBI-825 COH-29 G-207 TAS-1553	Gemcitabine has been approved for the treatment of cancer; Triapine, BBI-825, COH-29, G-207 and TAS-1553 are in clinical development.	In cancer, the expression and activity of RNR may undergo changes, and these alterations may be associated with cancer proliferation, metastasis, and drug resistance.	178, 179
IDH1/IDH2	TCA cycle	Olutasidenib Ivosidenib Enasidenib Mesylate	They have been approved for the treatment of IDH mutant cancers	IDH mutations are common in some cancers (glioma and acute myeloid leukemia). These mutations can produce 2-HG, which is carcinogenic. IDH1 may be a new target for pancreatic cancer	180-182
MDH	TCA cycle	FM-701	Preclinical data	The high expression of MDH1 and MDH2 is associated with poor prognosis and high invasiveness in some cancers.	183, 184
PGAM1	PPP	HK-B99 KH-3	HK-B99 in non-small cell lung cancer is in preclinical stage in China. The development of KH-3 has been discontinued.	PGAM1 is a potential drug target for the treatment of lung cancer	185, 186

HK, Hexokinase; PFKFB3, 6-phosphofructo-2-kinase; PKM2, pyruvate kinase M2; LDHA, lactate dehydrogenase A; GLUT1, glucose transporter 1; GLS/GLS1, glutaminase/glutaminase1; ASCT2, alanine-serine-cysteine transporter 2; CPS1, carbamoyl phosphate synthetase 1; FASN, fatty acid synthase; MAGL, monoacylglycerol lipase; AKT, protein kinase B; CPT1, carnitine acyl transferase 1; FAO, fatty acid oxidation; DHFR, dihydrofolate reductase; RNR, Ribonucleotide reductase; IDH1/IDH2, isocitrate dehydrogenase1/isocitrate dehydrogenase2; MDH, malate dehydrogenase; PGAM1, phosphoglycerate mutase 1.

**Table 3:** The current clinical trials and their results related to targeted cancer metabolism

Drug	Company	Type of drug	Target	Trial Phase	Results	Refs
Enhertu (trastuzumab deruxtecan)	Daiichi Sankyo and AstraZeneca	ADC	HER2	Approved for listing	Enhertu has received conditional approval from the National Medical Products Administration of China for the treatment of adult patients diagnosed with unresectable locally advanced or metastatic NSCLC characterized by HER2 activating mutations, provided that these patients have previously undergone at least one systemic therapy.	197
Trodelvy (sacituzumab govitecan)	Gilead Sciences	ADC	Trop-2	Clinical phase Ⅲ	Three indications have been approved in the US, and the TROPiCS-04 study is currently underway to evaluate the impact on overall survival in patients with metastatic or locally advanced urothelial carcinoma who are not candidates for surgical resection.	198
Jemperli	GlaxoSmithKline	Antibody	PD-1	Clinical phase Ⅲ	It has been approved by the FDA for the monotherapy of advanced or recurrent endometrial cancer with dMMR. Research is currently underway regarding the combination therapy with Zejula for the treatment of ovarian cancer.	199
Zejula	GlaxoSmithKline	Small molecule drugs	PARP	Clinical phase Ⅲ	As a PARP inhibitor, it can eliminate cancer cells by inhibiting the PARP-mediated DNA damage repair response. Research is currently being conducted on the combination therapy with Jemperli for the treatment of ovarian cancer.	199
Tiragolumab	Roche Holding AG	Monoclonal antibody	TIGIT	Clinical phase Ⅲ	The objective is to evaluate the efficacy and safety of first-line therapy combined with Tecentriq (a PD-L1 monoclonal antibody) in patients with locally advanced or metastatic NSCLC exhibiting high expression of PD-L1.	200
CUE-101	Cue Biopharma	Protein drugs	HPV E7	Clinical phase 1b	In combination with Keytruda, this research aims to evaluate the overall response rate as a potential first-line treatment for patients with HPV-positive recurrent or metastatic head and neck squamous cell carcinoma (R/M HNSCC).	201
Ipagratinib (ABSK011)	Shanghai Abbisko Biopharmaceutical Technology Co., Ltd.	Small molecule drugs	FGFR4	Clinical phase Ⅰ	It demonstrated controllable safety and excellent antitumor activity in AHCC characterized by FGF19 overexpression. The ORR and DCR were 44.8% and 79.3%, respectively.	202
Abivertinib (AC0010)	ACEA Pharma	EGFR TKI	EGFR	Clinical phase Ⅰ	In patients with EGFR gene-sensitive mutations who experienced progression after targeted therapy, the ORR was 38.9%, while the DCR was 73.0%.	203

HER2, human epidermal growth factor receptor 2; ADC, antibody-drug conjugate; NSCLC: non-small cell lung cancer; dMMR, DNA mismatch repair defect; PARP, poly ADP-ribose polymerase; AHCC, advanced hepatocellular carcinoma; EGFR, epidermal growth factor receptor; ORR, objective response rate; DCR, disease control rate
